# *Streptomyces* as a Prominent Resource of Future Anti-MRSA Drugs

**DOI:** 10.3389/fmicb.2018.02221

**Published:** 2018-09-24

**Authors:** Hefa Mangzira Kemung, Loh Teng-Hern Tan, Tahir Mehmood Khan, Kok-Gan Chan, Priyia Pusparajah, Bey-Hing Goh, Learn-Han Lee

**Affiliations:** ^1^Novel Bacteria and Drug Discovery Research Group, Biomedicine Research Advancement Centre, School of Pharmacy, Monash University Malaysia, Bandar Sunway, Malaysia; ^2^Biofunctional Molecule Exploratory Research Group, Biomedicine Research Advancement Centre, School of Pharmacy, Monash University Malaysia, Bandar Sunway, Malaysia; ^3^Jeffrey Cheah School of Medicine and Health Sciences, Monash University Malaysia, Bandar Sunway, Malaysia; ^4^The Institute of Pharmaceutical Sciences (IPS), University of Veterinary and Animal Sciences (UVAS), Lahore, Pakistan; ^5^Division of Genetics and Molecular Biology, Institute of Biological Sciences, Faculty of Science, University of Malaya, Kuala Lumpur, Malaysia; ^6^International Genome Centre, Jiangsu University, Zhenjiang, China; ^7^Center of Health Outcomes Research and Therapeutic Safety (Cohorts), School of Pharmaceutical Sciences, University of Phayao, Mueang Phayao, Thailand

**Keywords:** *Streptomyces*, methicillin-resistant *Staphylococcus aureus*, antibiotics, anti-MRSA, Actinobacteria

## Abstract

Methicillin-resistant *Staphylococcus aureus* (MRSA) pose a significant health threat as they tend to cause severe infections in vulnerable populations and are difficult to treat due to a limited range of effective antibiotics and also their ability to form biofilm. These organisms were once limited to hospital acquired infections but are now widely present in the community and even in animals. Furthermore, these organisms are constantly evolving to develop resistance to more antibiotics. This results in a need for new clinically useful antibiotics and one potential source are the *Streptomyces* which have already been the source of several anti-MRSA drugs including vancomycin. There remain large numbers of *Streptomyces* potentially undiscovered in underexplored regions such as mangrove, deserts, marine, and freshwater environments as well as endophytes. Organisms from these regions also face significant challenges to survival which often result in the production of novel bioactive compounds, several of which have already shown promise in drug development. We review the various mechanisms of antibiotic resistance in MRSA and all the known compounds isolated from *Streptomyces* with anti-MRSA activity with a focus on those from underexplored regions. The isolation of the full array of compounds *Streptomyces* are potentially capable of producing in the laboratory has proven a challenge, we also review techniques that have been used to overcome this obstacle including genetic cluster analysis. Additionally, we review the *in vivo* work done thus far with promising compounds of *Streptomyces* origin as well as the animal models that could be used for this work.

## Introduction

Methicillin-resistant *Staphylococcus aureus* (MRSA) show resistance to almost all therapeutic β-lactams and other classes of antibiotics. MRSA was first reported in 1961 (Jevons, 1961), when a β-lactamase producing strain of *S. aureus* which had previously been methicillin sensitive, developed methicillin resistance. The fact that this occurred only a year after the introduction of the semi-synthetic penicillin was truly a harbinger of the specter of drug resistance that would haunt healthcare providers in the years to come. MRSA has since been isolated in many hospitals around the world and currently represents a serious healthcare problem. It is particularly prevalent (>50%) in South America, Romania, and Japan and is becoming increasingly widespread in other countries (Lee et al., 2018). Concerns have also been raised over the emergence of MRSA among livestock due to the extensive use of antibiotics to prevent and treat infections (Conceição et al., 2017). Recently, cases of MRSA have been reported outside of the hospital settings, mainly affecting young, healthy individuals (Braun et al., 2016; Braun and Kahanov, 2018).

While MRSA generally do not cause severe disease, there are limited therapeutic options available to MRSA infections making all infections, even mild ones, noteworthy. By definition, MRSA are resistant to penicillin-like antibiotics, and they have now been noted to be developing resistance to other existing classes of antibiotics (Kaur and Chate, 2015). There is a constant hunt for new antibiotics but in the last few decades, only a limited number have been added to the clinician's arsenal; among them are linezolid in 2000 (Lee and Caffrey, 2017), daptomycin (a lipopeptide) in 2003 (Frankenfeld et al., 2018) and ceftaroline in 2010 (Long et al., 2014). Presently, vancomycin remains the most important first-line therapy for severe MRSA infection. However, the emergence of MRSA with reduced susceptibility to vancomycin (Ghahremani et al., 2018) as well as daptomycin (Roch et al., 2017) and linezolid resistance (De Dios Caballero et al., 2015) have been reported. Given that bacteria naturally evolve toward developing resistance to all antibiotics they are exposed to, there is a critical need for research focusing on the search of novel antibacterial agents as well as innovative approaches to combat MRSA. In light of the pressing need for new anti-MRSA drugs, the World Health Organization has also included MRSA as an important antibiotic-resistant bacteria requiring the urgent need for new drugs (WHO, 2017).

Natural sources such as microbes, plants, and animals have contributed immensely to the development of current drugs (Gu et al., 2013; Tang et al., 2016; Ma et al., 2018; Tan et al., 2018). Among these natural sources, microbes, particularly those belonging to the gram-positive Actinobacteria phylum, stand out as a rich source of drugs (Bérdy, 2012). The genus *Streptomyces* is categorized under the phylum Actinobacteria (Waksman and Henrici, 1943); they currently represent the most widely studied genus under the Actinobacteria phylum with 843 species and 38 subspecies to date (LPSN, 2018). The vast diversity within this genus based on its sheer numbers is particularly evident when compared with other genera: *Micromonospora* genus has 84 species and 7 subspecies, *Propionibacterium* has only 16 species and 4 subspecies, while *Salinispora* has 3 species (LPSN, 2018) at the time of writing (June 2018). Based on historical evidence, *Streptomyces* seem to be a viable target in the hunt for new drugs as they represent the source of 75% of clinically useful antibiotics presently available (Janardhan et al., 2014). One of the newer antibiotics currently in use, daptomycin, represents the latest contribution of *Streptomyces* in the fight against pathogenic microbes—it was discovered in the 1980s and approved by the US Food and Drug Regulatory Administration (US FDA) for clinical use in 2003 (Frankenfeld et al., 2018). To date, *Streptomyces*-derived daptomycin remains the only naturally produced antibiotic of a novel class introduced since 2003 and it is currently considered a first-line drug for treatment of MRSA bacteremia (Choo and Chambers, 2016).

*Streptomyce*s has a large genome, which logically contains many biosynthetic gene clusters (Bentley et al., 2002; Ikeda et al., 2003), a further indication of their potential ability to produce large numbers of compounds with diverse biological activities. However, under traditional culture condition, only a few compounds have so far been isolated—far less than what is expected based on the genome. Various methods discussed later are being used to overcome this problem. An additional recent concern is that analysis of *Streptomyces* from terrestrial soil has limited yield of new compounds but instead leads to rediscovery of known compounds. To overcome this problem, researchers now focus on isolating *Streptomyces* from underexplored ecosystems (Hong et al., 2009). Researchers are also attempting to utilize current genetic tools to identify gene clusters of promising compounds. Once identified, these biosynthetic gene clusters are modified in order to improve the efficacy of the compounds, produce better analogs of compounds, or increase the yield of the compound of interest (Alexander et al., 2010; Yang et al., 2017). Besides that, *in situ* computer-models have been used successfully to determine mechanism of action of some promising anti-MRSA compounds isolated from *Streptomyces*. Newer animal models have also been used to determine *in vivo* efficacy and toxicity of anti-MRSA compounds produced by *Streptomyces*. This review aims to highlight the potential of *Streptomyces* as a resource to combat MRSA—we look at all the anti-MRSA compounds derived from *Streptomyces* since the 1990s. We also discuss the ecological niches where the source organisms may be found, mechanism of actions of anti-MRSA compounds produced by *Streptomyces* and newer interventions for MRSA infection.

## Epidemiology of MRSA

Hospital-acquired MRSA (HA-MRSA) is now among the most problematic bacterial infections to treat (Kaur and Chate, 2015) and, alarmingly, it is responsible for about 20–80% of hospital infections (Krishnamurthy et al., 2014). Even though the incidence of HA-MRSA is reported to have reduced by 54.2% in the USA (Dantes et al., 2013), statistics from other parts of the world indicate this is not the general trend. For example, studies in South Africa, India and Pakistan revealed 52, 54.8, and 50% of hospital-acquired infections, respectively, were attributed to MRSA (Laxminarayan et al., 2013). According to the European Centre for Disease Prevention and Control (ECDC), the number of cases of MRSA infection varies greatly between the northern and southern regions of Europe. For example, Norway and Iceland were reported to have the lowest case of MRSA infection of 1.2 and 1.3%, respectively. Romania and Portugal represent the opposite end of the spectrum with the highest rate of cases reported with figures of 50.5 and 43.6%, respectively, in regard to invasive infections (ECDC, 2016). Most often, in hospital settings, MRSA occurs as a secondary infection and is most prevalent among the elderly, post-surgical and immunocompromised patients (Krishnamurthy et al., 2014). These secondary infections lead to increased healthcare costs, resulting from prolonged hospital stay and additional antibiotics (Nelson et al., 2015). In Japan alone, a recent comprehensive comparative cost analysis for MRSA has been estimated to be greater than all other non-MRSA infection (Uematsu et al., 2017). As an indication of the scale of the threat posed by these organisms, in the USA, MRSA kills more people than HIV and TB combined (Boucher and Corey, 2008). In hospitals, controlling the spread of MRSA is further complicated by their ability to form biofilms on the surfaces of medical devices. These biofilms tend to be resistant to disinfectants and may act as reservoirs for growing MRSA colonies that can be transferred to another host (Suzuki et al., 2015). The carriage rate in hospitals is estimated to be around 20–60% (Pathare et al., 2016) with the resultant implication that a large proportion of health care workers (Shibabaw et al., 2013; Khanal et al., 2015; El Aila et al., 2017) and patients (Ho and Hong Kong intensive care unit antimicrobial resistance study (HK-ICARE) Group, 2003; Aslam et al., 2013; Moyo et al., 2017) are carriers of MRSA. The most common way MRSA enters a host is through a breach in the skin (Datta et al., 2014) and tends to develop into an infection when there is immunodeficiency in the host. As a result of its tendency to affect more vulnerable patient populations, MRSA has not only become a difficult disease to treat but also a costly one. While immunocompromised individuals are at higher risk of MRSA infection, worryingly there have been recent reports of MRSA infection among healthy individuals, especially children (Davoodabadi et al., 2016).

About 20 years after the first reported case of MRSA, the organism was confirmed to have spread beyond the hospital environment to the community. The earliest report of community acquired MRSA (CA-MRSA) was in Detroit, Michigan, USA in 1980 (Saravolatz et al., 1982). Several other community-based infections were reported not long after—in the community of native Indians (Taylor et al., 1990), then in 1989-1991 it was found among the Aborigines of Western Australia (Udo et al., 1993) and after that it was detected in Europe (Stegger et al., 2014). While HA-MRSA cases seem to be on the decline in the USA, CA-MRSA has emerged more strongly in communities around the world. Currently, 2 out of 100 people are carriers of CA-MRSA (CDC, 2016); which is particularly worrying as CA-MRSA can more easily spread than HA-MRSA. It has been suggested that there may be a more mobile genetic element in CA-MRSA as compared to HA-MRSA (Udo and Boswihi, 2017; Boswihi and Udo, 2018). According to CDC, a CA-MRSA is categorized as such if infection is evident on admission or a MRSA culture was obtained within 48 h of admission, with no history of admissions or medical treatment requiring invasive procedures (Gorwitz et al., 2006).

Carrier status is not limited to humans as there is evidence to suggest that animals have also become carriers of MRSA, thus creating the possibility of spread of MRSA from animals to humans. The steady increase in the global population has resulted in ever increasing demands for food supply. As part of the efforts to increase the yield of livestock production, antibiotics have been increasingly used to prevent infection. However, the uncontrolled use of antibiotics has encouraged the development of antibiotic resistance including the emergence of MRSA within these animals (Van Boeckel et al., 2015). These livestock (Conceição et al., 2017) and their products tend to become reservoirs of MRSA (Asiimwe et al., 2017). Cases of animal-associated MRSA transfer to humans have been reported (Loncaric et al., 2013; Van Duijkeren et al., 2015). A few cases where MRSA was recovered from free-living animals (Wardyn et al., 2012; Porrero et al., 2013) and pets (Bierowiec et al., 2016) have been reported. This additional reservoir of MRSA in animals in the community creates an additional threat to public health.

## The mechanisms of antibiotic resistance in MRSA

The notion that “there is a pill for every ill” has led to a widespread public perception that medication of some sort is necessary to cure all forms of illnesses. A particularly pertinent example is the use of antibiotics for virtually any infection—including mild bacterial infections that do not warrant antibiotic treatment and even viral infections. This practice is a major cause of the rapid rise of antibiotic resistant bacteria. As a countermeasure, many countries have imposed tight regulations on the use and sale of antibiotics; however even in these countries, prescribers were found to overprescribe antibiotics. Given this scenario, it is unsurprising that in countries with less stringent regulations, there is a tendency to abuse antibiotics. This overuse of antibiotics in humans and animals has accelerated development of antibiotic resistance (Ventola, 2015).

Antibiotic resistance in and of itself is actually a natural phenomenon forming part of bacteria's inbuilt machinery to help them to adapt to new and changing environments. Soil bacteria possess an inbuilt “*resistome gene”* that helps them express resistance mechanisms in response to external events (Nesme and Simonet, 2015). Resistome genes present in soil bacteria can be horizontally transferred to pathogenic bacteria over a period of time. In the presence of antibiotics, these organisms also tend to develop resistance through an antibiotic resistance gene (Nesme and Simonet, 2015). This demonstrates that antibiotic resistance is inherent in bacteria and underlines the need for constant research to help develop a new supply of effective antibiotics in order to treat infections, including MRSA that are resistant to almost all β-lactam antibiotics. However, in order to effectively develop new therapies, it is crucial to first understand the various mechanisms of drug resistance and the elements of the bacterial cells that are new potential targets for drug development.

### MRSA mechanism of resistance to β-lactams

There have been studies exploring the origins of the methicillin resistance gene in MRSA. Studying sets of MRSA isolates revealed one single clone (Kreiswirth et al., 1993), with the most probable ancestral source dating back to *Staphylococcus sciuri*. Yet another study found support for *S. fleurettii* as the most probable origin (Tsubakishita et al., 2010). The study by Rolo et al. (2017) provided support that the Staphylococcal Chromosomal cassette (SCC*mec*), a mobile genetic element, evolved from three *Staphylococcus* species—*S. vitulinus, S. fleuretti*, and *S. sciuri*. Since its original development, the antibiotic resistance gene remains highly conserved in chromosomes of MRSA, and is currently used as a marker for screening and identifying MRSA isolates (Koupahi et al., 2016; Luo et al., 2017). Up to the present, 11 types of SCC*mec* (40-60Kb) have been identified (IWG-SCC, 1999). In the relevant types, the SCC*mec* is the region which contains the *mec*A gene which is responsible for the expression of PBP2a—an altered penicillin binding protein which has a low affinity for β-lactam antibiotics. On the basis of genetics, it is the expression of PBP2a that differentiates MRSA from other *S. aureus* strains, and confers resistance to most of β-lactams and other classes of antibiotics. The expression of PBP2a encoded on *mecA* gene is regulated by genes identified as *mecR1* and *mecI* on the *mecA* element, inducing and repressing transcription of PBP2a protein (Lee et al., 2018). Among the 11 SCC*mec* types (Mkrtchyan et al., 2015), 5 are known to be epidemic (Rachman et al., 2017).

*S. aureus* strains usually have 4 PBPs (PBP1, PBP2, PBP3, and PBP4) which play an important role in the biosynthesis of peptidoglycans—the structural frame of cell walls in gram-positive bacteria. As the name suggests, peptidoglycans are chains of glycans, namely *N*-acetylglucosamine (NAG) and *N*-acetylmuramic acid (NAM), cross-linked by peptides (Peacock and Paterson, 2015). Two biochemical reactions involved in cell wall synthesis are transglycosylation and transpeptidation, which are carried out by the enzymes transglycosylase and transpeptidase (PBP2), respectively. The former enzyme catalyses the elongation of glycans while PBP2 follows up from this action, crosslinking glycans at β1-4 NAM via 5 glycine amino acids (Fishovitz et al., 2014). This is key to our discussion as PBP2, or transpeptidase, is the target site of β-lactam antibiotics. In methicillin-sensitive *Staphylococcus aureus* (MSSA), the β-lactam class of antibiotics target the PBP2 causing cell death of susceptible *S. aureus*. In MRSA, however, the resistance-conferring PBP2a is overexpressed in the presence of β-lactams resulting in the cells still being able to generate sufficient cross-linking to survive.

The peptidoglycan layers are much thicker (20–80 nm) in gram-positive bacteria as compared to gram negative (1.5–10 nm; Mai-Prochnow et al., 2016), giving rise to the thick cell walls characteristic of gram positive bacteria. Peptidoglycans in gram-positive bacteria give shape and protect the cell from osmotic pressure and subsequent rupture and leaking of contents (Lovering et al., 2012). The β-lactam group of antibiotics prevent formation of this thick protective peptidoglycan layer by preventing cross linking of the glycan chains. Unfortunately, MRSA have developed resistance to almost all the β-lactam groups of antibiotics because SCC*mec* is a mobile element, it can be either horizontally transferred or vertically transferred (Grumann et al., 2014; Howden et al., 2014). The SCC*mec* types I, IV and V are the smallest of the 5 types and only express resistance toward the β-lactam groups of antibiotics; they therefore remain susceptible to other classes of antibiotics (Zuma et al., 2017). CA-MRSA have been found to contain smaller *mec*A IV and V genes compared to HA-MRSA, thus conferring the ability to move their mobile genetic elements much faster and with greater ease between various *S. aureus* chromosomes forming the basis of CA-MRSA's tendency to spread rapidly and more readily. This is demonstrated by the fact that cases of CA-MRSA are known to be widespread in Europe, USA, and other continents (Tavares et al., 2010; Dukic et al., 2013; San Sit et al., 2017). As for HA-MRSA, they possess SCC*mec* variants (SCC*mec* types II and III) which are larger in size, and thus providing additional genetic capacity which increases the likelihood of the presence of transposons and resistance genes (Hiramatsu et al., 2013). A specific region on the SCC*mec* known as the J region was shown to have antibiotic resistance genes of other classes of antibiotics (IWG-SCC, 1999).

### Resistance to other antibiotics

MRSA have been shown to have begun developing resistance to several important classes of antibiotics which are being used for treatment of severe MRSA infection including vancomycin, daptomycin, linezolid, tigecycline, and ceftaroline. Furthermore, MRSA strains were found to be resistant toward aminoglycosides, tetracyclines, lincosamides, and streptorubin B. This overwhelming spectrum of resistance imposes a huge burden on healthcare institutions. There are a few common mechanisms of resistance that pathogenic bacteria can develop when they exposed to antibiotics. The three main resistance mechanisms currently known are the inactivation of antibiotics by enzymes, efflux pumps that reduce intracellular drug concentration as well as alteration of target site. MRSA have been shown to employ all of these strategies.

To date, vancomycin remains the most important antibiotic for the treatment of severe MRSA infections. Vancomycin belongs to the glycopeptide antibiotic class that target the peptidoglycan layer of the bacterial cell wall. Hence, like β-lactam antibiotics, their effect is bactericidal. There appears to be a range of the level of resistance to vancomycin by *S. aureus* with the resistant organisms being either vancomycin-intermediate *S. aureus* (VISA) or vancomycin-resistant *S. aureus* (VRSA) with MIC ≤ 2 and 4–8 μg/μL, respectively (CLSI, 2015). The acquisition of vancomycin resistance in MRSA was shown to be different between the vancomycin resistant and the vancomycin intermediate strains. The vancomycin-resistant MRSA was found to contain the *vanA* operon (*vanA, vanH, vanY, vanX, vanZ*) and can either be acquired from vancomycin-resistant enterococci (VRE) via transposon 1546 or through horizontal transfer of original VRE plasmid, consequently leading to an alteration of the precursor of cell wall peptidoglycan, specifically the depsipeptide D-Ala-D-Lac. On the other hand, the emergence of vancomycin-intermediate MRSA involves chromosomal point mutations of polygenes resulting in the thickening of the peptidoglycan cell walls (McGuinness et al., 2017).

The second most important anti-MRSA drug is daptomycin which belongs to the lipopeptide antibiotic class (Schriever et al., 2005). Daptomycin targets the cell membrane causing depolarization and destabilization of the cell membrane leading to bactericidal effect (Alborn et al., 1991). The mechanism of resistance is multifactorial and involves a stepwise mutation of multiple genes (Bæk et al., 2015). According to Cafiso et al. (2014), *dltABCD* genes is a common pathway for reduced susceptibility of daptomycin, whereas *mprF* gene mutation was expressed in only certain strains that were tested (Cafiso et al., 2014). Another yet important gene mutation that was related to reduced daptomycin susceptibility is the RNA polymerase subunits (*rpoB*) (Cui et al., 2010). It is important to note that the incidence of daptomycin resistance is rare with absence of outbreak to date. Studies have demonstrated that this may be due to the involvement of high fitness cost in the development of daptomycin resistance required for dissemination (Roch et al., 2017).

Tigecycline is the first semi-synthetic antibiotic under the minocycline antibiotic class. Studies have reported MRSA to have overexpression of efflux pump through the mutations of both *mep*R and *mep*A genes resulting in overexpression of *mep*A and derepression of *mep*R. Furthermore, studies have shown the mutation of the ribosomal protein at S10 (Argudín et al., 2018). Study by Dabul et al. (2018) found out that mutation in *rpsJ* was not observed in their MRSA strain studied, but only the efflux mechanism was determined (Dabul et al., 2018). Interestingly, tigecycline resistance is not associated with fitness cost as compared with daptomycin (Dabul et al., 2018).

Ceftaroline belongs to the cephalosporin antibiotics and has been recently approved by FDA. Unlike other β-lactam antibiotics that target cell wall of *S. aureus*, ceftaroline has a high affinity toward the PBP2a of MRSA (Saravolatz et al., 2011). The ceftaroline susceptibility is defined as MIC of ≤ 1mg/L, intermediate resistance at MIC of 2 and ≥ 4 mg/L (CLSI, 2018). According to Alm et al. (2014), ceftaroline resistance is developed firstly by a mutation at the non-penicillin binding site of PBP2a and followed by a mutation at the active site (Long et al., 2014).

Antibiotics that inhibit protein synthesis are aminoglycosides, tetracyclines, macrolides, clindamycin, oxazolidones, and rifampin. Aminoglycoside resistance occurs via enzymatic inactivation—the most predominant aminoglycoside modifying enzyme is the aminoglycoside acetyl transferase responsible for aminoglycoside resistance in MRSA (Mahdiyoun et al., 2016). Tetracycline resistance in MRSA also occurs through a variety of mechanisms, including drug efflux and ribosomal protection mediated by *tetK* (Ullah et al., 2012) and *tetM* (Ong et al., 2017), respectively. Macrolide resistance in MRSA appears to be related to methylation of ribosomes by enzymes encoded on erythromycin resistance methylase (*erm*) genes. Studies have shown that MRSA strains express either dominant *ermA* (Lim et al., 2012) or *ermC* genes (Da Paz Pereira et al., 2016; Osman et al., 2015;), which is common among macrolide, lincosamide and streptogramin B (MLS_B_) resistant organisms. Linezolid belongs to the oxazolidone class of antibiotics and is a protein synthesis inhibitor (Kloss et al., 1999) with bacteriostatic action. The resistance gene responsible for linezolid resistance was identified as *cfr* gene, encoding a methyltransferase that modifies the 23S rRNA site of the 50S ribosomal subunit, preventing linezolid to bind to it (Toh et al., 2007; Quiles-Melero et al., 2012). Work on these organisms reported the presence of *cfr* gene in chromosomes of clinical human MRSA isolates as being responsible for mediating resistance in MRSA (Morales et al., 2010). Rifampin is a first-line drug for treatment of TB which is also used clinically to treat severe MRSA as an adjunct to vancomycin. Studies identified the *rpoB* gene as conferring resistance to rifampin by point mutation in the conserved region for ß-subunit of RNA polymerase (Van Rensburg et al., 2012).

Fluoroquinolones belong to the quinolone antibiotics that target DNA gyrases involved in bacterial DNA synthesis (Hooper and Jacoby, 2016). Resistance in MRSA appears to be mediated through a combination of mechanisms such as alteration of target site and prevention of drug access to the bacterial cell by efflux pump. NorA is one of the multidrug efflux pumps identified in MRSA. This pump belongs to major facilitator superfamily (MFS) which extrudes quinolone compounds. An additional mechanism of quinolone resistance in MRSA is the alteration of topoisomerase IV which is the primary target site for quinolone. This appears to be predominantly the result of a mutation occurring in the quinolone resistance determinant region (QRDR) of *parC* gene (Hashem et al., 2013; Hooper and Jacoby, 2016).

## Quorum sensing in MRSA

Quorum sensing is the means through which bacteria sense the external environment in order to adapt to changes or stress and these may include pH, antibiotics, minerals Abisado et al., 2018; Igarashi et al., 2013) and even cell population density (Rutherford and Bassler, 2012). Regulating these changes helps bacteria to survive in critical conditions. The two-component signaling (TCS) system called histidine kinase sensor and its cognate response regulator (Utsumi, 2017) facilitate the regulation of changes that is required by bacteria in order to cope with the outside environment. To date, there are many TCSs found in bacterial populace (Fabret and Hoch, 1998; Lange et al., 1999; Kawada-Matsuo et al., 2013; Guo et al., 2017), of which however, the WalK/WalR system stand out as an important TCS for regulating cell wall metabolism (Zheng et al., 2015). Interestingly, it is so far found in gram-positive bacteria with low G + C content such as *S. aureus*. Among the 16 TCSs found in *S. aureus*, the WalK/WalR was observed to be single most important regulator for virulence and cell wall metabolism among others functions (Ji et al., 2016). In cell wall metabolism, the WalK/WalR system activates autolysins known as peptidoglycan hydrolases that facilitates restructuring of peptidoglycan layer of the cell wall and promotes continuous cell growth and division (Utsumi, 2017). Since the cell wall provides the structural support necessary for cell survival, the WalK/WalR system therefore plays a significant role in gram-positive bacteria such as MRSA. Further, WalK is a master regulator for cell wall metabolism as nine cell wall metabolism genes are dependent on WalK/WalR (Utsumi, 2017). Previous studies have shown that inhibiting the WalK/WalR system is detrimental and bactericidal to cells (Gotoh et al., 2010; Igarashi et al., 2013). Therefore, WalK/WalR system is a promising target for drug development of anti-MRSA therapy. Among other TCSs are those that regulate biofilm formation and virulence factors that may offer potential for future antibiotics to treat MRSA-related infections.

### Biofilm and virulence of MRSA

Biofilm refers to a community of microbes that is firmly attached to surfaces and surrounded by a matrix of biopolymers (Flemming et al., 2016). Even though biofilm was first proposed in the 1970's by Costerton (Costerton et al., 1978), the importance of biofilm only became more apparent recently when genes responsible for expressing biofilm were characterized (Cucarella et al., 2001; Atshan et al., 2012; McCourt et al., 2014). Biofilm is a pressing medical problem in hospital settings, especially with regards to HA-MRSA infections, as it facilitates the persistence of MRSA in hospitals. Biofilm confers bacteria protection from extracellular threats such as antibiotics, disinfectants and the human immune response. The biofilm environment also allows bacterial communities to communicate with each other through quorum sensing molecules, for nutrients and space. Furthermore, the transfer of antibiotic resistance genes within the biofilm is also possible. These factors result in these organisms within biofilms demonstrating 1,000-times resistance to normal antibiotic doses (Wu et al., 2015). The communities of microbes then grow and mature within the matrix. Once the organisms mature, they detach from the biofilm and seek a new residence to colonize where they once again begin the process of biofilm formation (Otto, 2013). In hospitals, their ability to form biofilms on the surface of medical devices is a major concern (Suzuki et al., 2015) as insertion of medical devices infected with MRSA into the human body are a reservoir of infection that is extremely difficult to treat. It has been estimated that about 65–80% of human infections with MRSA are associated to biofilm formation (Jamal et al., 2017).

Biofilm, along with adhesion, are in fact two important mechanisms identified so far for successful colonization of host tissue or artificial surfaces (Mirzaee et al., 2014), and researchers have identified genes and factors responsible for these. Proteins found on the surface of MRSA, particularly fibronectin binding proteins A (*fnbp*A) and B (*fnbp*B*)*, autolysin enzyme and extracellular DNA (eDNA) are largely responsible for adhesion and biofilm formation. Unlike biofilm in MSSA, biofilm in MRSA is dependent on fibronectin surface protein, for adhesion to surfaces and are encoded on the *fnbp*A and *fnbp*B genes. Other proteins implicated in adhesion are the clumping factors A and B encoded on *clf* AB genes, accumulation associated protein (*aap*) and protein A (SpA). The biofilm formation in MRSA is mostly proteinaceous matrix derived from extracellular DNA (eDNA) and *fnbp* (McCarthy et al., 2015). Special proteins observed in MRSA are responsible for lysing cells and providing eDNA for the structural component of biofilm. Furthermore, studies have shown that expression of *mec*A in MRSA isolates leads to marked repression of global accessory genes (*agr*) and subsequent reduction in expression of virulence genes (McCarthy et al., 2015) while an activation of the Staphylococcal accessory regulator gene (*sarA*) is observed. Because virulence expression of *agr* loci is relatively reduced in HA-MRSA compared to CA-MRSA, it is believed that they are less virulent in nature but remain active biofilm producers. This may well prove to be a survival strategy HA-MRSA use, especially in healthcare institutions. Developing drugs that target proteins and signaling molecules in biofilm formation may prove beneficial in addressing MRSA infections.

Virulence is the ability of harmful microbes to invade and colonize the human body and they do so by producing virulence factors- small molecules or structures (Allen et al., 2014). Virulence factors of MRSA vary in degree depending on the type of MRSA and the condition of the host. An important virulence factor is Panton-Valentine Leukocidin (PVL), first described by Panton and Valentine (1932) and is increasingly found in CA-MRSA (Grumann et al., 2014). Infections with organisms carrying PVL tend to progress from mild skin and soft tissue infection (SSTI) such as boils and cellulitis, to more severe invasive infection such as severe abscesses, necrotizing pneumonia and increased complications in pneumonia (Haider and Wright, 2013). PVL in association with other leukocidin protein LukS and LukF, enables MRSA to invade cells causing severe invasive infection (Zhang et al., 2018). Other important virulence factors secreted by MRSA are Staphylococcal enterotoxins serotypes A-Q(SEs), toxic shock syndrome toxin (TSST), cytolytic toxins (hemolysis), exfoliative toxins and enzymes causing food-poisoning related diarrhea and emesis, low blood pressure and shock, bleeding and red blistering of skin, respectively (Otto, 2014).

The alarming reports of MRSA cases emerging in hospitals, communities and animals combined with the limited availability of antibiotics to treat MRSA has made it a very important infectious disease globally. At present, vancomycin remains the most important first-line therapy for severe MRSA infection (Boswihi and Udo, 2018). In order to successfully seek novel antibiotics and adjuvant therapies to treat resistant infections, it is necessary to home in on promising techniques and sources. The current search techniques for interesting compounds from *Streptomyces* should make use of new genetic tools and chemistry to accelerate the search for new treatments of MRSA. In terms of sources, microbes particularly *Streptomyces* have been demonstrated to be prolific producers of new compounds in the past and seem to show promise to continue in the present. Compounds with anti-MRSA activity isolated from *Streptomyces* are summarized in Supplementary Table 1. It is hoped that by utilizing advancements of genetic and chemical technology, these anti-MRSA compounds from *Streptomyces* can be further improved to be able to be used in clinical settings.

## What are *Streptomyces*?

*Streptomyces* are filamentous gram-positive bacteria that are categorized under the phylum Actinobacteria (Waksman and Henrici, 1943). To date, there are about 843 species and 38 subspecies with validly published names in bacterio.net (LPSN, 2018) at the time of writing (June 2018). *Streptomyces* is known to be a very robust genus of bacteria in terms of their ability to thrive in inhospitable soil conditions. They have been isolated from soil samples from the richly biodiverse tropical regions to the far reaches of the Arctic Circle. The soil samples from these ecoregions seem to hold promising *Streptomyces* with interesting chemical and genetic make-up (Ser et al., 2015a, 2016b,c, 2017, 2018a) for drug discovery work. It was logical to begin a search for *Streptomyces* by investigating soil samples because soil is readily available and is known to be a rich source of microbes (Hibbing et al., 2010). Further, *Streptomyces* are saprophytic bacteria that thrives on dead and decaying materials. Most studies investigating the microbial diversity of soil samples have reported Actinobacteria, particularly *Streptomyces* as the predominant species. The fact that *Streptomyces* can be found in almost all places studied thus far, suggest that they are a highly competent bacterial species. On a molecular level, this demonstrates their superior genetic and metabolic potential that allows them to dominate the microbial population. To understand the mechanisms that led to the latter observation, researchers began studying related genes and proteins that could contribute to their versatility and ability to thrive. One of the interesting findings, which is now considered as a characteristic of *Streptomyces*, are their high guanine plus cytosine (G + C) genomic content. On average, they carry 70% G + C content which is considered very high as compared to 40% G + C content of *Bacillus subtilis* the model gram-positive bacteria (Kunst et al., 1997). In the most widely studied species *S. coelicor*, the G + C content measures up to 72.1% (Bentley et al., 2002). The high G + C content in *Streptomyces* species is believed to have accumulated over time by a selection process of adaptation to new environment; and is thought to have granted them overarching dominance in soil.

They also express powerful secretory systems constituting 40% of the ATP-binding cassette (ABC) transporters and MFS (Zhou et al., 2016). As a result, they have attracted interest from the biotechnology industry in the production of recombinant proteins. One example is the use of *Streptomyces lividans* as a potential producer for recombinant proteins due to the possession of better excretory system as compared to the traditionally used *Escherichia coli* (Anné et al., 2014). In terms of their ecological role, *Streptomyces* utilize these secretory systems, to regulate the intake of nutrients, expel toxins and secrete enzymes. These enzymes help break down tough plant and animal materials into soluble substances that can be easily taken up through their mycelia. Once in the mycelial cells, they are utilized for basic metabolic processes to produce ethanol, amino acids, nucleotides, organic acids and vitamins. Under stressful conditions, when soil nutrients become scarce, *Streptomyces* initiate a process called programmed cell death (PCD), whereby autolysin enzymes break down mycelial cells into amino acids and sugars, providing the building blocks for aerial hyphae that will eventually produce and carry spores. This process ensures that *Streptomyces* continue to grow, reproduce and survive under stressful conditions. A key point of interest from a drug development point of view is the biosynthesis of secondary metabolites that accompanies PCD. As the name suggests, secondary metabolites are derived from primary metabolites and cons diverse compounds with different biological activities (Ser et al., 2016b, 2018b; Tan et al., 2016). Some of these compounds are antibiotics and are today perceived to play an ecological role in terms of suppressing potential competitors by killing them or inhibiting their metabolic growth. This behavior becomes essential in protecting their food supply from other microbes and it demonstrates a metabolic pathway that is highly organized and well-co-ordinated, giving rise to their dominance (Ilic-Tomic et al., 2015). It is therefore not surprising to see that *Streptomyces* have a large genome size in order to facilitate production of the range of regulatory proteins and extracellular enzymes these processes require. Many of these secondary metabolites have been successfully used as antibiotics in treating infections in humans and animals (Cho et al., 2012), though resistance has now developed to many of them. The role of *Streptomyces* as an important antibiotic producer warrants their continued exploration as a promising source in the search for new anti-MRSA antibiotics.

*Streptomyces* first contributed to our antibiotic arsenal in 1940 with the discovery of the naturally produced antibiotic streptomycin from soil-derived *Streptomyces griseus* (Woodruff, 2014). Since then, 2 Nobel Prizes have been awarded to researchers for the study of *Streptomyces* (Woodruff, 2014; Dlugónska, 2015). *Streptomyces* have made invaluable contributions to conventional medicines (Janardhan et al., 2014) with 75% of antibiotics clinically used in humans having their origins in *Streptomyces* derived compounds while 60% have been used in animals (Cho et al., 2012; Ser et al., 2015b; Law et al., 2017a,b).

Although artificial synthesis of new drugs is theoretically promising, natural sources such as microbes remain the better producers of antibiotics as they provide lead molecules for development of current antibiotics (Ser et al., 2016a). According to the review of Bérdy, the success rate of drugs produced through chemical synthesis compared to microbes is 0.005 to 1.6 (Bérdy, 2012). Even today, microbe-derived chemical compounds continue to inspire the development of new antibiotics (Newman and Cragg, 2016). For example, some of the newly approved drugs from the year 2000 onwards are actually synthetic analogs of antibiotics produced by *Streptomyces* namely tigecycline, everolimus, miglustat, daptomycin, biapenem, ertapenem, pimecrolimos, and ceftarolinefosamil (De Lima Procópio et al., 2012; Kumar and Chopra, 2013). Similarly, *Streptomyces* are also the source of some drugs that are currently undergoing clinical trials using combination formulation of cephalosporin-lactam (e.g., ceftazidime-avibactam and ceftolozane-tazobactam), a new cephalosporin siderophore S649266, omadacycline (phase 2 trial) and eravacycline (Fernandes and Martens, 2017). Fosfomycin was previously isolated from *S. fradiae* in 1969 and is currently undergoing comparative phase 3 clinical trials to evaluate its intravenous preparation against piperacillin/ tazobactam in the treatment of chronic urinary tract infection and acute pyelonephritis in hospitalized adults (Fernandes and Martens, 2017).

Over the years, the number of compounds reported from *Streptomyces* have significantly reduced, resulting in fewer drugs approved for clinical uses. A clear example is the US FDA approved drug daptomycin-the only new class of antibiotic introduced since 2003. Overall, *Streptomyces* have produced an estimated 10, 400 bioactive compounds since the discovery of streptomycin. The number of compounds has waxed and waned over the years—bioactive compounds isolated from *Streptomyces* increased from 2,900 between 1940 and 1974 to 5,100 compounds in the years 1975–2000, before dropping to 2,400 compounds in the period between 2001 and 2010 (Bérdy, 2012). The decrease seen was mainly attributed to rediscovery of compounds from *Streptomyces* isolated from soil, particularly from the land (Bérdy, 2012). Given that genome mining has demonstrated the potential of *Streptomyces* to produce many more compounds than what is currently observed, the search for new bioactive compounds from *Streptomyces* continued with focus on other understudied ecosystem. Researchers began to focus on understudied environments particularly those with harsh condition postulating that the challenge to survival in these situations is the driving force for speciation resulting in a rich diversity of microbiological sources including *Streptomyces* (Hong et al., 2009). In the following paragraphs, we focus on the various ecological sources of *Streptomyces* with anti-MRSA activity.

## Ecological sources of *Streptomyces* with anti-MRSA potentials

**Figure 1:**
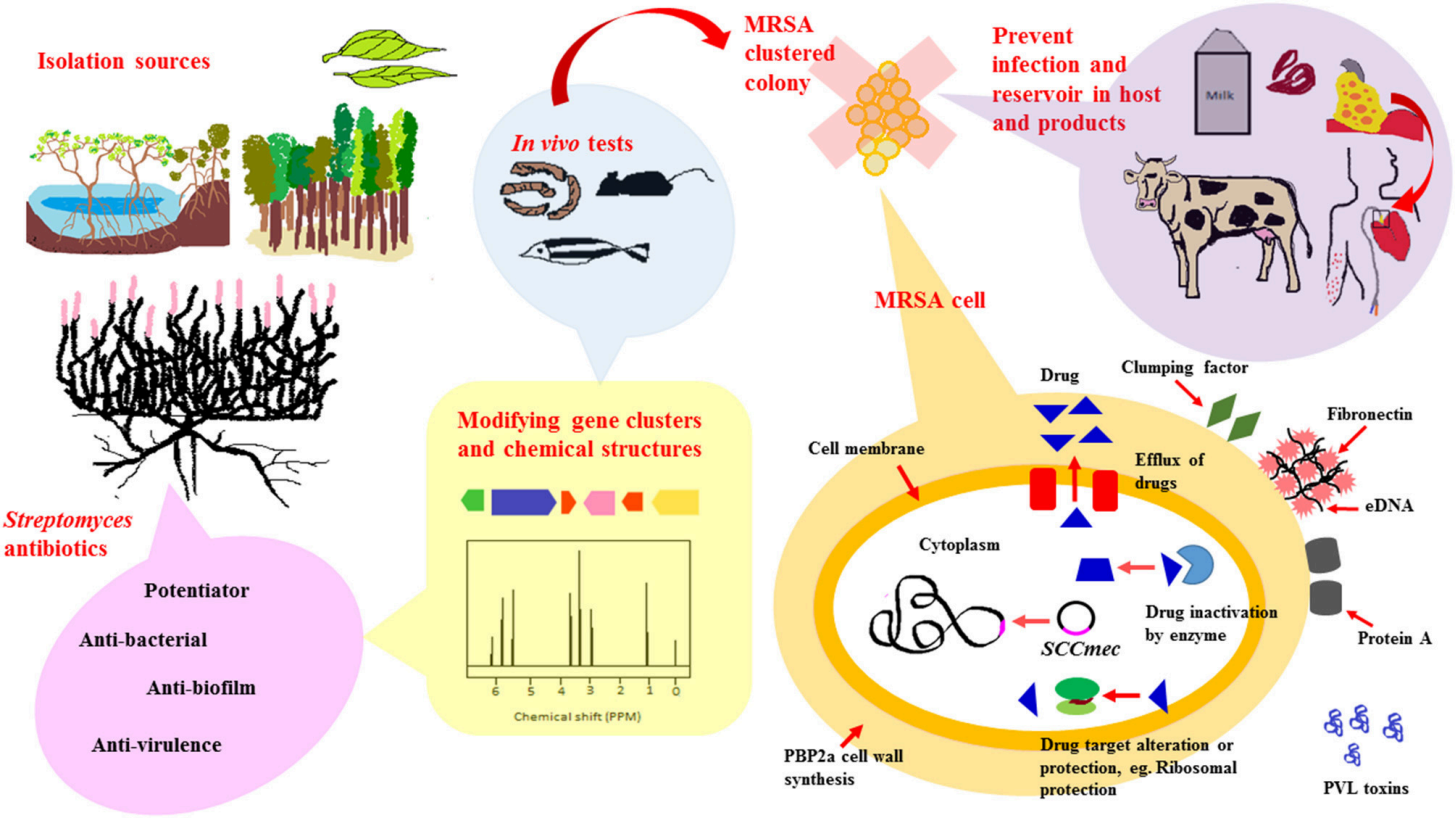
*Streptomyces* as potential sources for newer anti-MRSA compounds. To date, anti-MRSA compounds produced by *Streptomyces* have been isolated from various ecological sources that include terrestrial, marine, mangrove ecosystems and endophytes. *Streptomyces* are widely known for their ability to produce diverse range of antibiotic-like compounds. These compounds exert their anti-MRSA activity via different mode of actions, either by direct killing, synergistic or potentiator, anti-biofilm and anti-virulence properties. Recent advancement of technology, genetic and chemical modification, have facilitated production of antibiotics isolated from *Streptomyces* to form new chemical entities with improved anti-MRSA potency. These newly modified anti-MRSA compounds are further subjected to animal models-mice, silkworm and zebrafish, to validate their clinical efficacy prior to clinical trials. MRSA carry a mobile genetic element, Staphylococcal chromosomal cassette (*SCCmec*), that can be horizontally transferred from one bacteria to another. In addition to that, they are also capable of developing resistance mechanisms to non-β-lactam antibiotics, either via (i) Efflux of intracellular drug concentration (ii) Enzyme that inactivates drugs (iii) Altered drug target. Developing antibiotics that target these resistance mechanism, biofilm formation and virulence, would markedly reduce spread and infection of MRSA in hosts and animal products.

Based on existing literature, *Streptomyces* derived from numerous ecological sources are active producers of natural compounds that exhibit anti-MRSA activity. These ecological sources include soil collected from terrestrial regions such as tropical forests, marine regions encompassing marine sediments and symbionts as well as newer understudied ecological niches such as endophytes, freshwater, deserts and mangrove ecosystem. A schematic diagram shown in Figure 1 is included to give an overview of the extent of work in the area of *Streptomyces* as potential sources of anti-MRSA agents.

### Terrestrial soil

Literature analysis shows that prior to the year 2000, *Streptomyces*-based research mainly focused on terrestrial soil (Raja et al., 2003). The enthusiasm for sampling terrestrial soil *Streptomyces* for the search of newer anti-MRSA seems to have gained momentum around the 1990s. The studies presented in Supplementary Table 1 are reflective of published work undertaken from 1990 to the current year. In total, 86 *Streptomyces* species investigated have demonstrated promising anti-MRSA activity. From studies carried out so far, 37 of those promising *Streptomyces* strains isolated from terrestrial soil demonstrated moderate to potent anti-MRSA action. In Korea, the emergence of multi-drug resistant *S. aureus* (MDRSA) prompted the search for newer sources of anti-MRSA drugs. In an attempt to seek new treatment for the MRSA strains in their country, Lee et al. (1997) began investigating *Streptomyces* extracts from local soil against clinical isolates of MDRSA. Their work led to the successful isolation of *Streptomyces* sp. HW-003 from the soil of the primary mountain forest Gyebangsan (1,577 m), a region known to have soil which has a fine texture of organic matter (Yang et al., 2014). Work on HW-003 then yielded the active compound AMRSA1 which showed potent anti-MRSA activity at 0.01–0.1 μg/mL (Lee et al., 1997) and is by far the most potent anti-MRSA compound isolated from terrestrial soil samples. Its structure however, remains unknown to date. Other promising compounds isolated from terrestrial soil include polyketomycin.

### Marine environments

Natural product researchers have also begun to explore the marine ecosystem which clearly represents an alternative source for novel *Streptomyces* as it covers 70% of the earth's surface (Beygmoradi and Homaei, 2017); especially considering that 90% of the underwater life-forms are still awaiting discovery (Mora et al., 2011; Li, 2016; NOAA, 2018). A literature search from 1990 to the present year yielded reports indicating 42 *Streptomyces* strains from the marine environment with compounds showing moderate to potent anti-MRSA activity. This strongly suggests an increasing trend in discovery of anti-MRSA compounds over time from the marine ecosystem, particularly the marine sediments.

### Newer and underexplored ecological sources

Natural product researchers are now exploring understudied ecosystems for potentially new *Streptomyces* that display new chemistry and biological activity–these include that biodiverse environments such as the freshwater, endophytes, deserts and mangrove ecosystem.

#### Freshwater environment

Freshwater ecosystems are natural water runways such as rivers, lakes and streams except saltwater (IPCC, 2007). According to the literature, only 4 *Streptomyces* species have so far been isolated from freshwater and their compounds tested for anti-MRSA activity (Malik et al., 2008; Zhu et al., 2013). One of these was *Streptomyces* sp. MC004 which was isolated from an acidic coral mine drainage which produced angucyclic quinones including angumicynone B which showed anti-MRSA activity at MIC of 12.5 μg/mL (Park et al., 2014). *Streptomyces fulvissimus* MTCC7336 produced a high molecular weight glycopeptide with anti-MRSA activity determined by disk diffusion method (19.00 ± 1.0 mm).

#### Endophytes

Endophytes are microbes that live inside plant tissues. They are assumed to form mutual relationship with host plant by producing defensive compounds in order to ward off potential plant pathogens. To date, they remain an understudied source for new compounds (Gouda et al., 2016). Based on the literature, only 3 endophytic *Streptomyces* have so far shown anti-MRSA activity. Interesting compounds derived from endophytes were previously reviewed by Martinez- Klimova (Martinez-Klimova et al., 2017) who found that the majority of endophytes isolated under Actinobacteria phylum were *Streptomyces* (Martinez-Klimova et al., 2017). *Streptomyces* sp. SUK25 was isolated from the root sample of *Zingiber spectabile* in Malaysia, producing compounds cyclo-(tryptophanyl-prolyl) and chloramphenicol. *Streptomyces* sp. SUK06, was isolated from *Thottea grandiflora* in Malaysia on the basis of its medicinal use- wound healing, skin infection or curing fever. The careful selection of medicinal plants resulted in isolation of *Streptomyces* having significant antimicrobial activity with a zone of inhibition of 37 mm (Ghadin et al., 2008). There are also reports of marine *Streptomyces* endophytes with anti-MRSA activity, for example *Streptomyces sundarbabensis* WR1L1S8 from the *Ficus* species or the brown alga. Among the 22 marine alga it was found that *Streptomyces* from *Ficus* species demonstrated promising antimicrobial activity inclusive of anti-MRSA activity (Djinni et al., 2013). The work so far suggests the benefits of focusing the search for endophytes rather than randomly selecting plants. Thus far, medicinal plants and algal, particularly *Ficus* species have been shown to be potential sources of endophytes with promising anti-MRSA activity.

#### Desert

Recently, deserts have become interesting sources of sampling *Streptomyces* with anti-MRSA activity. Deserts are barren dry land with extremely high temperature, making it difficult for plants and other organisms to survive there (Smith, 2018). Microbes or any form of desert lifeforms therefore must have developed unique metabolic pathways to allow them to tolerate extreme levels of heat and temperature. *Streptomyces* sp. C34 which was isolated from the Chilean hyper-arid Atacama desert soil produced chaxamycins with potent anti-MRSA activity with MIC of 0.13 μg/mL-0.25 μg/mL (Rateb et al., 2011). We did note there is no current published work on *Streptomyces* with anti-MRSA activity from the Arctic. It is therefore suggested that apart from desert, researchers interested in anti-MRSA compounds from *Streptomyces* may also want to focus on the Arctic.

#### Mangrove ecosystem

Mangroves represent an additional untapped ecological niche which may play an important role in harboring organisms which may produce potential contributors for lead molecules for new anti-MRSA drugs (Ser et al., 2015c; Tan et al., 2015). Mangroves are a very interesting ecological niche because they lie at an interface of terrestrial and marine ecosystems, hence, the prospect of finding novel *Streptomyces* that are adapted to survive this environment is exciting. It is likely that the chemical ecology in this environment will be very different from that of terrestrial as well as marine environments. Furthermore, mangrove also represents a viable research platform as it encompasses 75% of tropical coastlines and 25% of the world's coastlines. Despite the well-documented flora and fauna, the microbial diversity of mangrove forest remains underexplored (Xu et al., 2009; Lee et al., 2014a,b; Zainal et al., 2016; Tan et al., 2017). Based on the literature, a few anti-MRSA compounds have been reported from the mangrove forests (Supplementary Table 1).

This demonstrates that these hitherto underexplored fields offer potential for future therapeutic drugs targeting MRSA. Some of these anti-MRSA compounds have been further studied for their mechanism of action and are highlighted in the following paragraphs.

## Anti-MRSA compounds derived from *streptomyces* and their mechanisms of actions

**Figure 2:**
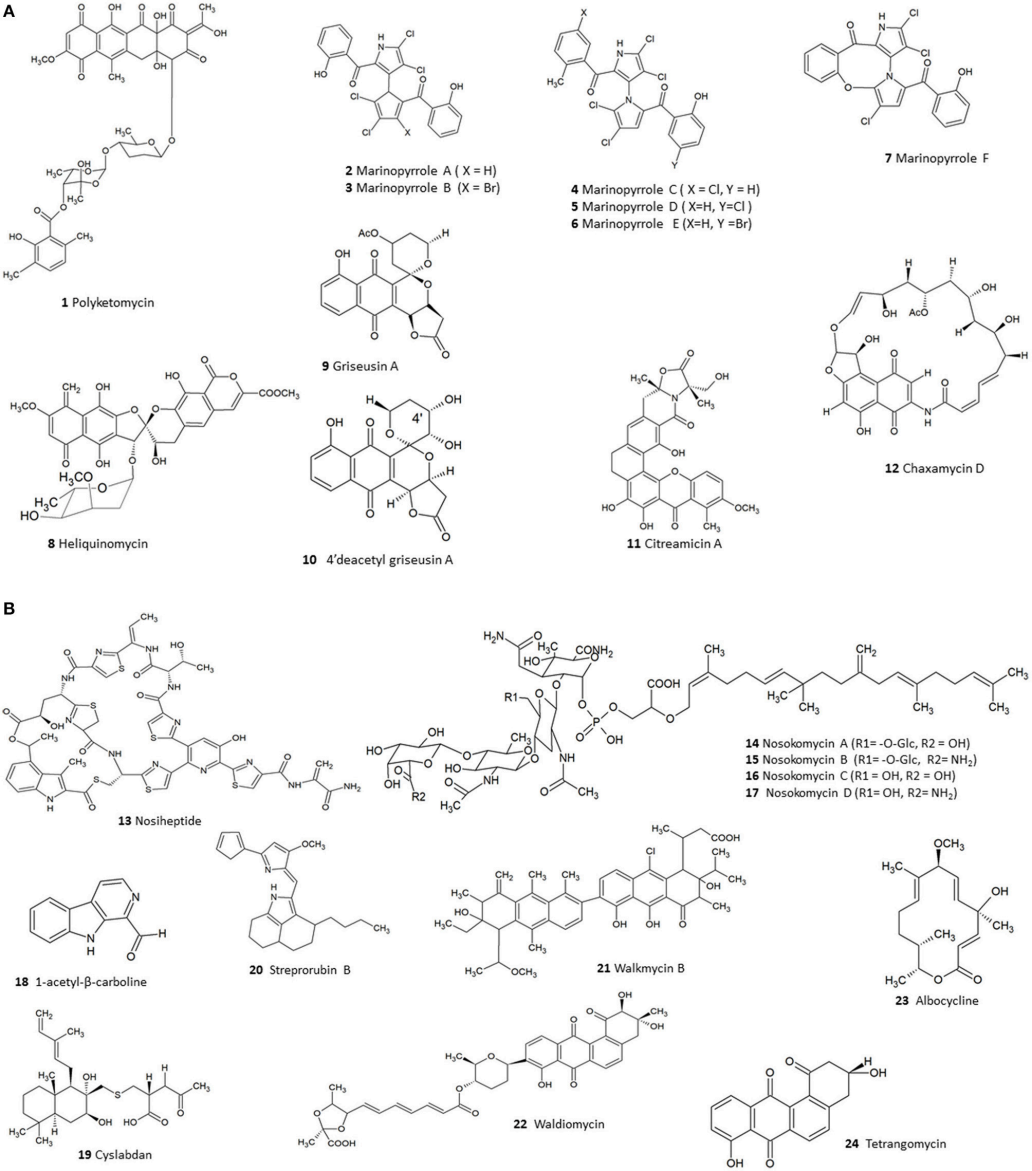
**(A)** The chemical structures of compounds isolated from *Streptomyces* with moderate to potent anti-MRSA compounds **(1–12)**. **(B)**. The chemical structures of compounds isolated from *Streptomyces* with moderate to potent anti-MRSA compounds **(13–24)**.

Existing literature so far have highlighted 124 compounds produced by *Streptomyces* which show moderate to potent anti-MRSA activity (Supplementary Table 1). A numerical analysis of these compounds shows that polyketides (PKS) form the largest group (53), the next being non-ribosomal peptides (NRPS) while others include smaller proportions of alkaloids, hybrids of PKS/NRPS and PKS/terpenoids. In fact, polyketide and NRPS biosynthetic pathways are the source of most *Streptomyces* derived conventional antibiotics; and logically these classes also constitute a large fraction of the substances demonstrating anti-MRSA activity. Among the molecules identified were several compounds—such as polyketomycin, heliquinomycin, griseusin A, 4′deacetyl griseusin, citreamicin θ A, chaxamycin D, nosiheptide, nosokomycin, and marinopyrrole A—which have been reported to be potent anti-MRSA compounds and exhibit lower MIC than several clinically used antibiotics such as vancomycin. Vancomycin has been used as a standard for testing of effectiveness of new compounds against MRSA (CLSI, 2015). The chemical structures of these bioactive compounds are depicted in Figure 2A **(1-12)**, Figure 2B **(13-24)**.

### PKS compounds with anti-MRSA activity

Polyketides (PKS) are common natural products among *Streptomyces* species and their synthesis is catalysed by enzymes known as polyketide synthase (PKS). These enzymes function together in a consecutive manner along the metabolic pathway. PKS shares a common pattern of biosynthetic steps with fatty acid synthase (FAS) (Jenke-Kodama et al., 2005), basically catalysing step-by-step condensation of simple carboxylic acids. Yet in PKS, additional modifications to structures are possible using specialized enzymes, different starter and extender units, reduction and cyclization reactions resulting in a wide array of antibiotic compounds with antibacterial actions. It is not surprising therefore, to find polyketides from *Streptomyces* with potent anti-MRSA activity such as polyketomycin.

#### Polyketomycin

The second most potent anti-MRSA compound known is polyketomycin (**1**) which was isolated from *Streptomyces* sp. MK277-AF1 found in a soil sample collected in the Kanagawa prefecture in Japan and has shown an MIC of 0.025–0.2 μg/mL (Momose et al., 1998). Polyketomycin (**1**) produced by *Streptomyces* was extensively studied and used as a model to identify gene clusters of potentially important antibiotics. The use of genomic mining has allowed the identification of biosynthetic gene clusters of antibiotic compounds from *Streptomyces. Streptomyces coelicolor*, the most extensively studied *Streptomyces* was found to potentially have 20 gene clusters (Bentley et al., 2002), indicating its potential for production of about 20 secondary metabolites.

Biosynthetic pathways of compounds that have shown promising biological activities are studied to identify their respective biosynthetic gene clusters. As such, polyketomycin as a polyketide is composed of a tetracyclic ring, 2 monosaccharides and a dimethyl salicylic acid. Based on the chemical structure, Paululat et al. (1999) postulated the biosynthetic gene clusters by firstly predicting the enzymes involved in the biosynthetic pathway. For example, the tetracyclic moiety and dimethyl salicylic acid moiety were assumed to derive from a PKS II and PKS I, respectively. The two sugar moieties, which are 6-deoxysugars, might have been synthesized from glucose via a pathway involving a TDP-glucose-4, 6-dehydratase during biosynthesis and attached by two glycosyltransferases. Once the gene clusters have been identified, alterations can be made to the natural pathways to create modified chemical structures with improved clinical efficacy using nature's biosynthetic machinery (Greule et al., 2017).

Polyketomycin (**1**) seems to be attractive not only because of its potent anti-MRSA activity and naturally high yield (4.3 g/L) but also because of the composition of its biosynthetic product. The polyketide synthase of the aglycone and the enzymes of the salicylic acid moiety combine different activities from known pathways of other strains in a new manner (Paululat et al., 1999). Hence, polyketomycin has been proposed to be a good target for combinatorial biosynthesis to modify its existing structure to increase its potential applications. Combinatorial biosynthesis utilizes genetic engineering to bring about changes to biosynthetic pathways of isolated compounds and hence, produce new structures (Floss, 2006).

Although polyketomycin was discovered in 1998, its mechanism of action has not been determined. It is however known that polyketomycin belongs to the anthracycline group of antibiotics which are already well-known for their anti-tumor properties. Current anticancer drugs doxorubicin, daunarobucin, and rubidazone are clinically used for treating cancer whose mechanism is defined by the inhibition of DNA polymerase. Hence, it is likely that polyketomycin belonging to anthracycline antibiotic can exhibit the same mechanism of action toward MRSA; this was suggested by another study investigating anti-malarial activity of polyketomycin (Otoguro et al., 2003). Recent discovery of other anthracyclines from *Streptomyces* include cervimycins and DMI-2 isolated from *Streptomyces* sp. 560 and *Streptomyces tendae* HKI-179, respectively. Interestingly the anthracyclines mentioned have a *p*-quinone D ring in common (Daum et al., 2009) which may be the element giving rise to the anti-MRSA activity observed.

#### Heliquinomycin

Heliquinomycin (**8**), a polyketide isolated from *Streptomyces* sp. MJ929 in Japan, exhibited low MIC of < 0.05–0.1 μg/mL (Supplementary Table 1). Interestingly, it was found to inhibit the human DNA helicase, a known target for tumor growth. Heliquinomycin belongs to the rubromycin family which are known for their activity against telomerase and retroviral reverse transcriptase which may have led to further investigation of anti-cancer instead of MRSA properties. To date, no separate mechanism of action has been afforded to heliquinomycin on MRSA (Chino et al., 1996, 1997, 1998).

#### Griseusin A and 4′deacetyl griseusin A

Aromatic polyketide griseusin A (9) and 4′deacetyl griseusin A (10) were isolated from *S. griseus* M33-5 found in the Turkish soil (Urgen et al., 2010). Griseusin A and 4′deacetyl griseusin belong to the griseusin antibiotics and demonstrate anti-MRSA activity of 1 and 0.5 μg/mL, respectively (Supplementary Table 1). Griseusin compounds represent a subclass of pyranonaphthoquinones and include griseusin A and B whose structures were initially characterized from *S. griseus* in 1976 (Tsuji et al., 1976). Griseusins are also known for their potent anticancer properties and are commonly found distributed among actinomycetes particularly *Streptomyces* and *Nocardiopsis* sp. (Ding et al., 2012) as well as fungi. For example, griseusins were previously isolated from *S. griseus* K-63, 3–5, *S. griseus* MJ361-48F3, *Streptomyces* sp. IFM 11307, actinomycete strain MJ932-SF3, *Nocardiopsis* sp. and *Penicillium* sp. Recent studies discovered newer griseusin E and 4′deacetyl griseusin A in *Streptomyces* IFM 11307 with 4′deacetyl griseusin shown to be the most effective to overcome tumor necrosis factor related apoptosis-inducing ligand (TRAIL) resistance. Their antibiotic activity and the fact that they are one of the complex pyranonapthoquinones, has prompted interest in their chemical synthesis. The first successful total synthesis of griseusin A was published in 1983 (Kometani et al., 1983) followed by synthesis of analogs of griseusin A (Brimble et al., 1999). Further interest into PKS of griseusin by *S. griseus* K-63 led to the identification of 5 *gris* genes that encode for griseusin. It is known that PKS type 2 (PKS II) compounds are mostly aromatic in nature. Yet the aromatic PKS of griseusin are found to be programmed differently from other aromatic PKS. This sparked renewed interest in these compounds and studies were carried out to identify programming mechanism of these 5 genes (Yu et al., 1994). However, the mechanism of action of these compounds with respect to their anti-MRSA activity is still elusive.

#### Citreamicin θ A

The citreamicin group of xanthone antibiotics (polycyclic aromatic antibiotics) were first isolated in 1989 from *Micromonospora citrea* in lake Manyara, Tanzania (Carter et al., 1990). Among the citreamicins isolated at that time were α, β, γ, ζ, and η. All four compounds shared a common xanthone nucleus and methoxy group at position at C17 except citreamicin η which has a hydrogen instead. All displayed anti-MRSA activity with citreamicin η being the most potent anti-MRSA compound with MIC of <0.015 μg/mL even though it lacked a methoxy group at position 17 (Carter et al., 1990). Yet another analog of citreamicin known as neocitreamicin I and its derivate neocitreamicin II were reported in 2008 with anti-MRSA MIC at 0.12–0.50 and 0.5–1.0 μg/mL, respectively. These neocitreamicin also lacked a methoxy group at C17 indicating that modification on C17 does not significantly affect their anti-MRSA activity (Peoples et al., 2008). The recent discovery of marine-derived citreamicin analogs citreamicin θ A (**11**), citreamicin θ B, citreaglycon A, and dehydrocitreaglycon A from *Streptomyces caelestis* isolated from the Red Sea exhibited potent anti-MRSA activity especially citreamicin θ A (**11**) and citreamicin θ B at 0.25 μg/mL. Furthermore, anti-MRSA activity of the compounds of citreamicin θ A (**11**) and θ B were assumed to be due to 5 membered ring group which was absent in citreaglycon A and dehydrocitreaglycon A (Liu et al., 2012). Citreamicin δ and ε were also isolated in 2008 from *Streptomyces vinaceus* from a soil sample obtained from a river. They were observed to be structurally similar to citreamicin η except for the relocation of a methoxy group but yet showed anti-MRSA activity in the range of ≤ 0.5–2 and 0.12–0.25 μg/mL (Hopp et al., 2008). Despite its potent anti-MRSA activity, its mechanism of action remains unknown at present time. However, it is speculated that the 5 membered ring confers citreamicin its potent anti-MRSA activity. Chemical synthesis has proven difficult to date since its discovery in 1989 with research only achieving a 11-step synthesis of the pentacyclic core of citreamicin η (Blumberg and Martin, 2017).

#### Chaxamycin D

Chaxamycin D **(12**) was originally isolated from *Streptomyces* sp. strain C34 from soil samples from the Chilean hyper-arid Atacama Desert (Supplementary Table 1; Rateb et al., 2011). Chaxamycin D (**12**) belongs to the ansamycin group of compounds, which also includes the potent anti-TB drug rifamycin. Even though its MIC is lower than 2 μg/mL, activity was compared to rifampicin having an MIC of 0.002–4 μg/mL. Ansamycins are widely known for their anti-tumor properties via inhibition of heat shock protein 90 (Hsp90) via selective interaction with the ATP-binding pocket in the *N*-terminal domain; and *in situ* molecular studies of chaxamycin demonstrated that it also possesses this property. Due to its promising anti-MRSA activity, further studies have been carried out to determine the gene cluster of chaxamycin, as was done with polyketomycin. However, this experiment used mutational analysis (mutasynthesis) in the natural producer and heterologous expression in *S. coelicolor* A3 (2) strain M1152. Restoration of chaxamycin production in a non-producing cxmK mutant was achieved by supplementing the growth medium with 3-amino-5-hydroxybenzoic acid (Castro et al., 2015). Mutasynthesis is a method that attempts to modify the structure of a known compound, by feeding the microbe with structural analog precursors with the aim to improve its pharmacological property. In a similar study carried out by Feng et al. (2014) mutasynthesis was used where *Streptomyces ansochromogenes* were fed with structural analog precursors of the anti-fungal compound nikkomycins. Although the nikkomycins produced through mutasynthesis was the same as the one produced in the natural host, the resultant showed better stability (Feng et al., 2014). Hence, mutasynthesis can be used to potentially improve the pharmacological properties of promising anti-MRSA compounds.

### NRPS compounds with anti-MRSA activity

NRPS include clinically important drugs such as daptomycin, bleomycin and cyclosporine. The synthetic pathway for NRPS compounds involves a system of enzymes that utilizes 500 non-protein building blocks apart from the 20 amino acids to form diverse linear and cyclic peptides with a wide array of biological activities (Strieker et al., 2010) through postranslational modification of peptides such as acylation, halogenation, or hydroxylation. Non-ribosomal peptides often have cyclic and/or branched structures and can contain non-proteinogenic amino acids including D-amino acids. From the literature reviewed (Supplementary Table 1), compounds AMRSA1 (Lee et al., 1997) and nosiheptide (Haste et al., 2012) showed anti-MRSA activity at 0.01–0.1 and ≤ 0.25 μg/mL, respectively. These 2 compounds are among the most potent anti-MRSA compounds known, having lower MIC readings compared to positive controls used. Given that the study on AMRSA1 is still limited, only nosiheptide is further discussed as follows.

#### Nosiheptide

Nosiheptide (**13**) was isolated from *Streptomyces* sp. CNT 373 isolated from marine sediments off the coast of Fiji (Haste et al., 2012), as part of the marine drug discovery project by Fenical and Jensen. This protein synthesis inhibitor (Cundliffe and Thompson, 1981), had a rapid bactericidal action in the first 6 h and a post-antibiotic effect (PAE) exceeding 9 h. In addition, its MIC level remained unaffected in the presence of 20% serum suggesting it may have promise in clinical settings. One of the surprising elements of nosiheptide is its lack of cytotoxicity. It is assumed that most of the compounds isolated from marine environments possess cytotoxic properties, relevant in anti-cancer drugs. After the discovery of nosiheptide (**13**) in 1970 under the name multhiomycin (Tanaka et al., 1970; Endo and Yonehara, 1978) it was approved for use as a growth promoter in animal feeds. Its anti-MRSA activity was only determined later on by Haste et al. (2012). The mechanism of action of nosiheptide is closely related to thiostrepton which acts on 50S ribosomes, however, thiostrepton shows a higher affinity than nosiheptide, with 30% and 70% inhibition of binding, respectively (Cundliffe and Thompson, 1981). Despite their similarity of mechanism of action, they differ in the biosynthetic pathways for the formation of the terminal amide group. While nosiheptide (**13**) carries an endogenous amide moiety, thiostrepton has its amide moiety incorporated by an asparagine synthase-like protein (Liu et al., 2015). Total synthesis of nosiheptide (**13**) was achieved by initial formation of a linear precursor and followed by macrocyclization to form the compound (Wojtas et al., 2016). Anti-MRSA activity was maintained after synthesis of nosiheptide (**13**). Successful total synthesis of nosiheptide paves the way to modify structures in order to derive newer antibiotics for clinical use. Nosiheptide has already proven to be effective in animal models (20 mg/kg) in a murine model, whereby intraperitoneal MRSA was treated with nosiheptide (**13**) with positive results (Haste et al., 2012).

### Nosokomycin B, A phosphoglycolipid compounds with anti-MRSA activity

Nosokomycins A (**14**), B (**15**), C (**16**), and D (**17**) were isolated from *Streptomyces* sp. K04-0144 found in the soil of Japan. The MIC with respect to MRSA for nosokomycins A- D was 0.125 μg/mL while vancomycin and imipenem were 0.5 and 16 μg/mL, respectively; indicating nosokomycin has more potent *in vitro* anti-MRSA activity than vancomycin (Supplementary Table 1; Uchida et al., 2010). In their subsequent investigation of nosokomycin's anti-MRSA activity against 54 MRSA strains, nosokomycin B (**15**) was clearly the most potent, followed by nosokomycins A (**14**), D (**17**), and C (**16**) (Uchida et al., 2014). The activity of nosokomycin has been studied in detail relating to its chemical structure; in terms of its structure-activity relationship, it was observed that the presence of a glucose residue at R1 and an amino residue at R2 was necessary for potent anti-MRSA activity. Nosokomycin belongs to a family of compounds known as moenomycins. Structurally, nosokomycin B (**15**) exists to be identical to a semisynthetic moenomycin A derivative, which lacks the chromophoric cyclopentenone moiety (Uchida et al., 2010). Moenomycin A possesses a glucose residue at R1 and a chromophoric cyclopentenone residue via an amide bond at R2, and shows weak bactericidal activity against MRSA. Even though the MIC of moenomycins against gram-positive bacteria were of submicrogram concentrations, there was one study on MIC of moenomycin A against MRSA, ATCC 43300 which showed an MIC of 4 μg/mL (Vancomycin = 1 μg/mL). Given that nosokomycin A (**14**) and B (**15**) are biosynthetic intermediates of moenomycin A, it was unexpected that while nosokomycins were isolated, no members of the moenomycins were isolated from producing *Streptomyces* strain K04-0144 (Uchida et al., 2010). The authors suggested that the genes for moenomycin A are lacking or not activated in the producing strain.

An interesting feature of moenomycins is their underlying mechanism of antibacterial activity. They seem to target the peptidoglycan glycosyltransferases directly, and they are the only natural products known to do so. This differs from vancomycin which targets the substrates for these enzymes and therefore acts indirectly. While vancomycin acts by competing with substrate *N*-acetylmuramic acid (NAM) and *N*-acetylglucosamine (NAG) for the enzyme, moenomycins directly bind to the enzyme itself. It was shown that nosokomycin B has submicrogram MIC concentration even lower than vancomycin. Moenomycins have great therapeutic potential as studies have shown that it is necessary to target the active site of the enzyme to achieve stronger therapeutic effect; and the work done so far supports that moenomycins bind to the active site of the enzyme (Uchida et al., 2010).

The moenomycin class have a unique structure and mechanism of action resulting in potent anti-MRSA effect, yet they do not make good drug candidates in their natural form because of their poor pharmacokinetic profile. The C25 lipid chain which is responsible for the potent MRSA of moenomycin is also the cause of its long half-life and low bioavailability. It was assumed that the long lipid chain helped the drug to anchor itself onto the cytoplasmic membrane. Modifying the lipid chain by decreasing the carbon chain was shown to improve pharmacokinetic properties but led to the loss of activity (Fuse et al., 2010; Galley et al., 2014). According to the literature reviewed, there are no reports of studies looking into the gene clusters for nosokomycin B as a means to improve its pharmacokinetic properties. This may be because the nosokomycins A and B were identified as intermediates of moenomycin biosynthesis. Therefore, the gene clusters for producing MmA can be used to improve the pharmacokinetics of both MmA and nosokomycin. Also, to date no pharmacokinetic study exists to determine its potential in human therapy–moenomycins are currently used only in veterinary settings (Uchida et al., 2010).

### Marinopyrrole A, an alkaloid with anti-MRSA activity

The research group by Hughes et al. (2010) isolated marinopyrroles with anti-MRSA activity from obligate marine *Streptomyces* sp. CNQ-418 which was in turn isolated from marine sediments at 51 m depth off the coast of La Jolla, California (Hughes et al., 2008, 2010). The 6 active compounds identified as marinopyrroles A-F (**2-7**) belong to the 1, 3′ bispyrroles alkaloid—they are densely halogenated and axially chiral metabolites that contain an uncommon bispyrrole. The need for sea water is essential in the biosynthesis of the dense halogenated backbone structure. These sets of compounds are the first naturally occurring 1, 3′ bipyrroles. Apart from their intriguing chemistry, they exhibit potent anti-MRSA activity with MIC <1 μg/mL. Marinopyrrole C exhibited the lowest MIC at 0.16 μg/mL followed by marinopyrrole A at MIC 0.31 μg/mL (Supplementary Table 1; Hughes et al., 2010). This group of compounds have attracted a lot of interest due to their structure, antibiotic and antitumor activities (Cheng et al., 2010). However, they are unsuitable for clinical use in their natural form as they are neutralized by 20% human serum (Haste et al., 2011b). Numerous chemical and genetic studies have since begun to work on ways to modify the structure to overcome this problem. For example, some of the studies focused on total synthesis of marinopyrroles A and B as well as their analogs (Nicolaou et al., 2011). The first total synthesis was carried out by Cheng et al. (2010) and the biosynthesis of marinopyrrole was successfully carried out by Yamanaka et al. (2012).

Marinopyrroles are the first naturally-occurring microbial compounds classified under the group of 1, 3′-bipyrroles and found to show potent antibacterial activity against MRSA (Hughes et al., 2008). These marine-derived compounds were produced from an obligate marine *Streptomyces* sp. strain CNQ-418 isolated from marine sediment. The considerable attention given toward this interesting group of compounds have also led to the successful chemical synthesis of a series of novel marinopyrrole derivatives (Hughes et al., 2010; Nicolaou et al., 2011) exhibiting promising biological properties including anti-MRSA activity (Haste et al., 2011b; Liu et al., 2014).

In 2008, marinopyrroles A (**2**) and B (**3**) were both isolated from the extract of *Streptomyces* strain CNQ- 418 in a seawater-based culture broth and their structure determined. Since then, marinopyrroles C- F (**4-7)** have been identified from *Streptomyces* under optimized culturing conditions. As the major metabolite, the antibacterial activity of marinopyrrole A (**2**) has been studied extensively against a variety of clinically important MRSA strains. It was observed that marinopyrrole A (**2**) showed potent concentration-dependent bactericidal activity against clinically relevant HA-MRSA and CA-MRSA strains (Haste et al., 2011b). Marinopyrrole A displayed substantial concentration- dependent killing against MRSA strain TCH1516 and was far more rapid in its antibiotic action than either vancomycin or linezolid. Using time-kill assay, marinopyrrole A at 10× MIC (3.75 μg/ml) showed a 2-log-unit kill of MRSA TCH1516 within 9 h, while the activity of vancomycin at 10 MIC (20 μg/ml) was much slower, reducing the initial inoculum by only about 10-fold. Treatment with marinopyrrole A at 20× MIC (7.5 μg/ml) reduced the initial inoculum by nearly 6- log-fold within 9 h. Moreover, marinopyrrole A (**2**) exhibits a prolonged PAE effect measures the effects of antibiotics at set times after exposure (Supplementary Table 1). This may be a more clinically significant measure than MIC which only demonstrates an all-or nothing relationship with a constant antibiotic concentration and therefore represents threshold concentration only. In this case, marinopyrrole A (**2**) at 1×, 10×, or 20× MIC exhibited a concentration-dependent PAE against MRSA strain TCH1516. A longer recovery rate of MRSA culture was noted with use of marinopyrrole compared to vancomycin and linezolid between 4 and 6 h at 20× MIC (Supplementary Table 1).

In terms of its clinical applications, marinopyrrole A (**2**) was shown to have affinity toward binding to plastic surfaces, allowing it to be applied as an antibiotic-lock agent which is relevant for the use as a topical agent or in local therapy of device-related MRSA infection. However, the study demonstrated the potential uses of marinopyrrole A (**2**) as a systemic antibacterial therapy may be hampered by its poor pharmacological profile in which its antibacterial efficacy was markedly reduced by serum. However, recent work has successfully modified structure of marinopyrrole and its derivatives resulting in retained or improved anti-MRSA activity and a reduced susceptibility to serum inactivation (Cheng C. et al., 2013; Liu et al., 2014). The overall evidence so far suggests that this group of unique natural products, marinopyrroles from marine origin *Streptomyces* bacteria, serve as important chemical structures for future modification and optimization in the continuous effort to develop new antibiotic therapy against MRSA.

## Combinatorial therapy/synergism: conventional antibiotics + new *Streptomyces* derived natural products

An alternative to developing new anti-MRSA compounds is to focus on improving the effectiveness of currently available antibiotics such as β-lactams. Since developing a totally new drug is costly and time-consuming, a more cost-effective approach would be to enhance the anti-MRSA activity of existing drugs as measured by a significantly reduced MIC indicating the bacteria become increasingly susceptible to the levels of drug concentration in blood and tissues.

Antimicrobial drug combination approaches aimed at improving the effectiveness of anti-MRSA drugs (reducing MIC) has become a potential area of research in drug discovery. The urgent need to have new antibiotics to eradicate MRSA infection has further validated this area of research, especially when MRSA is resistant to most of the β-lactam antibiotics. Potentiating or enhancing the effectiveness of existing antibiotics through synergism studies has been used to identify new and better combinations with enhanced ability to eradicate infections. Synergism is a favorable effect observed in antimicrobial combination studies whereby activity is significantly greater when two agents are combined than that provided by the sum of each agent alone. Synergy is more likely to manifest when the ratio of the concentration of each antibiotic to the MIC of that antibiotic was same for all components of the mixture (Jain et al., 2011).

The rationale for combination of antibiotic therapy for bacterial infections in general is that it enhances activity, reduces toxicity, may prevent the emergence of resistance or even enable treatment of a polymicrobial infection. The checkerboard is a useful method for studying synergism since a number of combination concentrations can be prepared and studied via broth 2-fold dilution with synergism detected as no visible growth (Stein et al., 2015). According to the Clinical Laboratory Standards Institute guidelines for broth microdilution, the MIC was defined as the lowest concentration of antibiotic that completely inhibited the growth of the organism as detected with the naked eye (CLSI, 2015).

### 1-acetyl-β-carboline and penicillins

*Streptomyces sp*. 04DHS2 was isolated from marine sediment and was shown to produce the alkaloid 1-acetyl-β-carboline. Used alone, 1-acetyl-β-carboline (**18**) exhibited an MIC of 64 μg/mL (Supplementary Table 1). However, when used in conjunction with several other penicillins using the checkerboard method, 1-acetyl-β-carboline (**18**) showed synergistic action based on the fractional inhibitory concentration (FIC) index (Shin et al., 2010). The study showed synergism between the combinations of 1-acetyl-β-carboline (**18**) with either ampicillin or penicillin against 14 MRSA strains (Shin et al., 2010). Given that MRSA have become resistant to the penicillin group of antibiotics, the study also revealed the potential of the synergistic effect of 1-acetyl-β-carboline (**18**) in restoring the antibacterial property of β- lactams against MRSA.

### Cyslabdan and carbapenems

Besides the checkerboard method, comparison between the MIC of the antibiotic when used alone and in combination was also performed to investigate the synergistic effects of the potentiating agent. Such was the case for investigating the effectiveness of cyslabdan as a potentiator of β-lactams. Cyslabdan **(19)** is described as a non-antibiotic compound belonging to the labdan-type diterpene. It was isolated from *Streptomyces* sp. K04-0144 and shown to enhance the antibacterial activity of carbapenems against MRSA K24 (Supplementary Table 1). Among the carbapenems, the anti-MRSA activity of imipenem was synergistically enhanced the most when combined with cyslabdan. Cyslabdan (**19**) exhibited weak anti-MRSA activity (MIC of 64 μg/ml) and no effect on MRSA at 10 μg/ml. When in combination with cyslabdan (**19**), the anti-MRSA activity of imipenem activity was enhanced with a reduction in MIC from 16 to 0.015 μg/ml (Fukumoto et al., 2008). This has large potential impact on health economics as carbapenems like imipenem, being the most potent of the β-lactams, have a high production cost, making the drug even more expensive to purchase. The cost of treatment could be significantly reduced if the effective dose of imipenem needed can be reduced with the combination use of a potentiator. These findings also prompted further studies on the mechanism of action via which cyslabdan (**19**) potentiates the anti-MRSA activity of imipenem (Koyama et al., 2012). The study showed that cyslabdan (**19**) appears to be the first known inhibitor to FemA enzyme, which is involved in pentaglycine interpeptide bridge formation during the synthesis of peptidoglycan. Therefore, the biosynthetic pathway of pentaglycine interpeptide bridge formation was suggested to be a potential target for rendering MRSA susceptible toward β-lactams.

## Anti-biofilm and anti-virulence activities

Biofilm formation is a key virulence determinant of MRSA; creating drugs able to induce disruption in the process of biofilm formation or its structure has become a potential area of research. This is because biofilm protects the MRSA from biocidals such as antibiotics and the immune system. Compounds from *Streptomyces* such as streptorubin B (**20**) have shown promising results in disrupting biofilm formation. Streptorubin B was originally isolated from *Streptomyces* sp. strain MC11024, and even though its MIC was 32 μg/mL, which is considerably higher than the vancomycin standard MIC of ≤ 2 μg/ml it demonstrated potential as a biofilm inhibitor by reducing the biofilm of MRSA to only 27% at 1 μg/mL (Suzuki et al., 2015). Since streptorubin B (**20**) was tested against one strain of MRSA, that is MRSA 315, increasing the number of strains tested will further demonstrate its inhibitory effect against MRSA biofilm. Streptorubin B (**20**) belongs to the previously discovered prodiginine class of pigmented antibiotics, known for their anti-tumor (Kojiri et al., 1993; Nakajima et al., 1993) and immunosuppressant actions (Songia et al., 1997) and now it has also shown potential as an anti-MRSA agent. Naturally occurring prodiginines have been isolated from actinomycetes, *Serratia, Pseudomonas*, and *Vibrio* among other bacterial species (Gerber and Lechevalier, 1976). From the limited data available, it seems justified to explore streptorubin B further to establish its role as an anti-MRSA agent.

A recent study showed that the ethyl acetate extract from *Streptomyces* sp. SBT343 significantly reduced the biofilm formation of several Staphylococcal species including USA300 (MRSA) at the maximum inhibitory concentration of 125 μg/mL. However, it did not display an inhibitory effect on the bacterial growth of *Staphylococcus* species as well as gram-negative *Pseudomonas aeruginosa*; overall indicating that it has selective anti-biofilm activity against Staphylococcal bacteria (Balasubramanian et al., 2017). Aside from high selectivity, no clinically relevant levels of cytotoxicity have been demonstrated, making this a promising source for an anti-biofilm against Staphylococcus species. Biofilms tend to complicate the therapeutic regime leading to increased duration of treatment, whereby the drug is required to penetrate through the biofilm to reach its target site. Most often, this can lead to treatment failure and consequently death. This has become a concern when treating severe MRSA infection with mainline drugs such as vancomycin. Using additional antibiotics with vancomycin, has been shown to dramatically improve the efficacy of vancomycin in both *in vitro* (Pistella et al., 2005) and *in vivo* mouse model (Shi et al., 2014). Given that vancomycin-intermediate and vancomycin-resistant *S. aureus* (VISA and VRSA) strains have been reported in some parts of the world, the need to develop new antibiotics is critical.

### Quorum sensing inhibitors

Apart from drugs targeting the formation and structure of biofilm, cell wall metabolism is another new focus of research for novel antibiotics. The WalK/WalR pathway is the main signaling pathway in low G + C content gram-positive bacteria such as MRSA and is responsible for cell wall metabolism and biofilm formation. It is assumed that inhibiting WalK/WalR consequently causes cell death. Studies investigating the potential of *Streptomyces* as producers of WalK/R inhibitors identified walkmycin B (**21**) (Okada et al., 2010) and waldiomycin (**22**) (Igarashi et al., 2013) as potent (0.39 μg/mL) and moderate (16 μg/mL) anti-MRSA agents, respectively.

The quorum sensing pathway offers a new potential target of new anti-MRSA compounds. Since quorum sensing plays a vital role in biofilm formation, it can be used as a potential drug target. In fact, studies have been carried out screening *Streptomyces* extracts that can inhibit the quorum sensing pathway in some gram positive and gram-negative bacteria (Hassan et al., 2016; Miao et al., 2017).

### Attenuation of virulence factors

Newer strategies to overcome MRSA infection involves attenuation of virulence factors rather than direct inhibition of the cell. Compounds isolated from *Streptomyces* such as albocycline (**23**) (Reusser, 1969) were recently shown to selectively block cell wall synthesis in *S. aureus* (Koyama et al., 2013). Recently identified metabolic pathways unique to *S. aureus* present alternative potential drug targets in combating antibiotic resistance. For example, the newly established biosynthetic pathway of heme—an essential iron carrying molecule—unique to gram-positive bacteria (Dailey et al., 2015). In addition, dehydrosqualine desaturase (CrtM) which is responsible for catalysing the first step in the synthesis of the yellow pigment staphyloxanthin (a virulence factor for *S. aureus)* may represent a potential target for new therapies. Studies provide evidence that *S. aureus* strains that produce staphyloxanthin are more resistant toward the host's immune system. Inhibition of this enzyme prevents the synthesis of staphyloxanthin. Using *in situ* docking models, the natural compound tetrangomycin (**24**) produced by *Streptomyces* sp. CAH29 was demonstrated to bind to the dehydrosqualene synthase enzyme (Özakin et al., 2016).

## *In vivo* evidence of anti-MRSA activities of *Streptomyces*

The *in vivo* stage of drug development involves the use of animals to investigate the safety and efficacy of new drugs in living mammalian organisms prior to clinical trials. This ensures that drugs are reasonably safe and effective before they are tested in human beings. However, prior to animal testing, promising drug candidates should, of course, demonstrate at least reasonable *in vitro* performance in terms of key parameters including MIC, rapid bactericidal activity and PAE (Guo et al., 2016).

These *in vivo* studies are necessary, because a compound that shows great potential *in vitro*, may not actually be effective in a biological system (Haste et al., 2011a,b). Unlike *in vitro* testing, in an actual biological system, a drug must first reach the target site where they can bind to and carry out their actions in order to have an effect; hence the pharmacokinetic processes of absorption, distribution, metabolism and excretion greatly influence their effectiveness and distinguishes the findings from *in vitro* and *in vivo* studies. Given that these drugs are usually intended ultimately for human use, it is important to select a model that mimics humans as closely as possible for the *in vivo* studies (Rosenthal and Brown, 2007).

Nosiheptide, which showed great promise *in vitro*, was tested *in vivo* on a murine model of intraperitoneal MRSA infection, where the test mice were given intraperitoneal injections of nosiheptide (20 mg/kg)—only 1 out of 10 infected mice died at the end of the study as compared to the controls where 6 out of 10 died on day 1. This provides evidence of significant *in vivo* activity of nosiheptide warranting further study (Haste et al., 2012). *In vivo* models are also used to determine toxicity of compounds isolated. Several of these compounds mentioned in Supplementary Table 1 are heliquinomycin, vinylamycin, polyketomycin, methylsulfomycin, lactinomycin, and gargantulide. Some of these compounds tested indicated toxicity in mice model, rendering them non-viable. Other examples include gargantulide isolated from marine *Streptomyces* which demonstrated a potent MIC of 2 μg/mL *in vitro*, methylsulfomycin which (at 25 mg/kg) was inconclusive due to precipitation formed while polyketomycin exhibited acute toxicity.

Moreover, the *in vivo* model selection needs to bear in mind several factors—mainly cost, time taken, and ethical considerations (Ferdowsian and Beck, 2011). Mammalian models tend to be more expensive and require a longer study period. Additionally, it is no longer possible to use mammalian models for most preliminary drug trials as the animal protection Act has tightened regulation of use of animal in experiments especially in European Union countries in 1998, where it is forbidden to use healthy animals in *in vivo* models (Uchida et al., 2014).

To overcome these problems, researchers have focused on non-mammalian animals such as Zebrafish, *Caenorhabditis elegans, Drosophila melanogaster* and silkworms as alternative hosts for *in vivo* screening systems. According to the literature reviewed in this paper (Supplementary Table 1), only silkworm and zebrafish have so far been employed for the screening of anti-MRSA compounds produced by *Streptomyces*. Uchida et al. (2014) demonstrated that a silkworm model could be successfully used in primary screening of new anti-MRSA agents—in this case, 4 potent anti-MRSA agents, nosokomycins A-D (MIC 0.125 μg/mL) (Supplementary Table 1) which were isolated from *Streptomyces*. Silkworm larvae infected with MRSA alone exhibited a lifespan of <3 days, compared to a survival rate of 3 days with vancomycin or other effective drugs including the nosokomycins (Uchida et al., 2014); the team also performed agar diffusion assay and also used a mouse model to provide supporting evidence of the reliability of the silkworm assay. The authors suggest that using a silkworm model as an *in vivo*-mimic assay may provide a more reliable prediction of actual *in vivo* activity compared to the traditional disk diffusion method. Hence, alternate animal models such as silkworms can become potential as alternate animal models in drug discovery and development of new anti-MRSA antibiotics.

Zebrafish embryos are an alternative model which is becoming increasingly popular in drug discovery work; they were initially introduced back in the 1970s but only recently became a model of choice in research on areas of genetically mutated diseases such as cancer. The advantages of this model are that they are cheap compared to rodents, they share 80% of genes with humans, and are much faster compared to trials on rodents. The zebrafish produce 200 eggs/week and so provides a reasonable source for research and they are easy to maintain. Additionally, the embryos are transparent making it easier to see the effects of test drugs on their organs and tissues. Zebrafish embryos were used to demonstrate the anti-MRSA activity of C23 (a purified compound from *Streptomyces rubrolavendulae* ICN3) as MRSA-infected zebrafish embryos survived with minimal toxicity effects in the presence of purified molecule C23 at 10 μg/ml (Kannan et al., 2014).

## Future directions/strategies

*Streptomyces* remain one of the most promising natural producers of antibiotics. Given the problems in rediscovery of known antibiotics from *Streptomyces*, it has been suggested that there may be a need to activate silent antibiotic gene clusters which may have gone dormant. This is possible with newer methods available such as: (1) The addition of signaling molecule *N*-acetylglucosamine in culture media of *Streptomyces clavuligerus, Streptomyces collinus, Streptomyces griseus, Streptomyces hygroscopicus*, and *Streptomyces venezuelae* have led to the activation of genes responsible for antibiotic productions. However, the same study also proved that this method does not work in all *Streptomyces* species (Rigali et al., 2008). In another study, Imai et al. (2015) showed that when *Streptomyces* are grown in the presence of lincomycin at sub-inhibitory concentration (1/12 or 1/3 its MIC) they produce compounds initially not produced in lincomycin-free media (Imai et al., 2015). (2) Similar results were seen with the addition of bacterial hormone gamma-butyrolactones (γ-butyrolactones), that serve as the main signaling molecules in *Streptomyces* species for the regulation of antibiotics by binding to cytoplasmic γ-butyrolactone receptors, whereby γ-butyrolactone induced gene expression of target genes and production of antibiotics in *Streptomyces* (Takano, 2006; Horinouchi and Beppu, 2007). Apart from γ-butyrolactones, the other 2 major groups of signaling hormones are the furans (e.g., 2-alkyl-4-hydroxymethylfuran-3-carboxylic acids) (Corre et al., 2008) and gamma-butenolides (γ-butenolides). In 2011, Kitani et al. (2011) was able to show that the γ-butenolide avenolide was responsible for stimulating the production of avermectin in *Streptomyces avermitilis* (Kitani et al., 2011). (3) Co-culture techniques are a powerful method of enhancing the biological activity of *Streptomyces* by allowing them to grow with another microbe in the same media (Sung et al., 2017). Sung et al. (2017) successfully used this method to enhance the anti-MRSA activity of extracts from marine *Streptomyces* sp. PTY087I2 M when grown in the presence of MRSA. The MIC was significantly reduced from 50 to 12.5 μg/mL, when grown under traditional culture condition and in co-culture system, respectively. These new approaches open possibilities to discover interesting anti-MRSA compounds from *Streptomyces* bacteria.

Also, understanding the biosynthetic pathways of important antibiotics is gaining increasing importance in the drug discovery process. A decade ago, cloning and identification of biosynthetic gene clusters was time-consuming and genome sequencing was expensive. Today, however, fast genome sequencing and genome mining have accelerated the identification of gene clusters–and knowledge of gene clusters provides crucial insights into modifying already existing antibiotics in order to improve them (Greule et al., 2017). These compounds may be effective in their ecological role to ward off competitors, but may need further modification in order to be viable antimicrobials in the human biological systems in terms of pharmacokinetics and dynamics. Apart from the new methods already mentioned in the paper having led to improved activity or increased yield, there are also reports of other approaches used recently to promote discovery of new compounds from *Streptomyces*. There are also reports of new methods of co-transformation carried out in *Streptomyces* sp. (Kallifidas et al., 2012). Despite the use of whole genome sequencing to identify the gene clusters, heterologous expression of these gene clusters often end up remaining silent. To overcome this problem, Kallifidas et al. (2012) used environmental DNA clones (eDNA), particularly those containing PKS type transcription factors and introduced them into *S. albus* harboring its corresponding minimal PKS containing clone. This method ultimately led to the production and isolation of tetarimycin in *S. albus*. In another example, Zhao et al. (2018) used a gene editing tool called the Clustered Regularly Interspaced Short Palindromic Repeats-Cas9 (CRISPR-Cas9) to introduce *kasO* promoter (97 base pair) successfully in *Streptomyces roseosporus* NRRL 15998. This promoter region then allowed for the activation of silent *aurR1* gene cluster, which when expressed, led to the production of auroramycin (Zhao et al., 2018). This was only possible after the biosynthetic gene cluster of auroramycin (*aurR1*) was identified (Zhao et al., 2018). These methods can further improve already known anti-MRSA compounds from *Streptomyces* and also allow for discovery of unknown compounds that are currently not produced because of the fact that these genes remain silent under traditional culture condition.

Given that antibiotic resistance is an inherent property of bacteria, the need to develop newer anti-microbial agents over time is inevitable. However, it is vital to understand that abuse and misuse of antibiotics have greatly accelerated the development of antibiotic resistance. As such, stewardship programs play a key role in creating greater public awareness of the importance of compliance to antibiotics, consequences of overprescribing and sanitary practices. Efforts to reduce the development of antibiotic resistance will help preserve the effective lifespan of last resort antibiotics that are still effective against MRSA such as vancomycin.

## Conclusion

In conclusion, MRSA poses a serious healthcare threat especially with the emergence of vancomycin resistant strains. *Streptomyces* have historically been and continue to play an important role as a source of new antibiotics—including compounds active against MRSA. Given the emerging trend of MRSA across the globe, the search for better therapeutic agents should be exploring all available resources—including *Streptomyces* from underexplored territory which are likely to yield exciting new compounds. This seems a promising approach since studies have shown that ecological variation provides a more suitable approach to accelerate the discovery rate of promising antibiotics. In this review, a number of new ecological niches have been discussed. Our review also revealed a spectrum of compounds isolated from *Streptomyces* with anti-MRSA activity and summarized the extent of studies so far carried out on these compounds.

Future work should focus on utilizing newly introduced genetic and chemical tools such as genome sequencing and mining and combinatorial biosynthesis to help create new and more effective antibiotics to address the emergence of antibiotic resistant infectious bacteria such as MRSA. Additionally, potential areas of research that remain yet to be undertaken have also been mentioned. The authors hope that future work can be focused on those underexplored niches, isolating new compounds and utilizing new technologies—genomic mining, combinatorial biosynthesis, mutasynthesis, to improve pharmacological properties of promising anti-MRSA compounds and thereby increase the number of successful anti-MRSA drugs for the treatment and halt spread of MRSA infection.

## Author contributions

HK, LT-HT, TK, K-GC, PP, B-HG and L-HL performed the literature search, data analysis as well the manuscript writing. Technical supports and proofreading were contributed by PP, B-HG, and L-HL. L-HL and B-HG founded the research project.

### Conflict of interest statement

The authors declare that the research was conducted in the absence of any commercial or financial relationships that could be construed as a potential conflict of interest.
