# Supplementary material for: Streptomyces as a Prominent Resource of Future Anti-MRSA Drugs
Source: Front Microbiol. 2018 Sep 24;9:2221. doi: 10.3389/fmicb.2018.02221 (PMC6165876; doi:10.3389/fmicb.2018.02221)
Supplement: Supplementary file 1 [file Data_Sheet_1.docx]

Table S1. Strains of *Streptomyces* sp. derived from different environments with potentials in production of anti-MRSA bioactive compounds.

| **Strain** | **Isolation source (Country)** | **Compound** | **Class** | **Anti-MRSA activity** | **Reference** |
| --- | --- | --- | --- | --- | --- |
| **PKS** | | | | | |
| *Streptomyces* sp. MJ47-72F6 | Terrestrial (Japan): Soil | Aldecalmycin | Dialkyldecalin | MIC (μg/mL) by agar dilution: 12.5μg/mL | (Sawa et al., 1994) |
| *Streptomyces* sp. MC004 | Freshwater (Korea): Acidic coral mine drainage | Angumicynones A (1); Angumicynones B (2);  Angucyclinones analogues compounds 3-8 | Angucyclic quinones | MIC (μg/mL):  Compound 1: NA;  Compound 2: 12.5μg/mL;  Angucyclinone analogues compounds 3-8: Not active | (Park et al., 2014) |
| *Streptomyces sundarbansensis* WR1L1S8 | Marine (Algeria): Endophyte from *Ficus* sp., a brown algae | Compound 13:  [2-hydroxy-5-((6-hydroxy-4-oxo-4H-pyran-2-yl) methyl)-2- propylchroman-4-one];  Phaeochromycin B (7); Phaeochromycin C (9); Phaeochromycin E (11) | Phaeocromycin | Agar diffusion and MIC (μg/mL):  Compound 13: < 2μg/mL;  Phaeochromycin B (7): NA;  Phaeochromycin C (9) and  Phaeochromycin E (11): Not active | (Djinni et al., 2013) |
| *Streptomyces sundarbansensis* WR1L1S8 | Marine (Algeria): Endophyte from *Ficus* sp., a brown algae | Initial test with ethyl acetate extract; Polyketide [2-hydroxy-5-((6-hydroxy-4-oxo-4H-pyran-2-yl) methyl)-2-propylchroman-4-one] | NA | Agar cylinder method: 17.23 ± 0.71mm | (Djinni et al., 2014) |
| *Streptomyces* sp. OUCMDZ-1703 | Marine (China): Unknown soft coral | Strepchloritide A (1) and strepchloritide B (2); | Strepchloritides | Method NA:  Compounds 3 and 5 with ≥ 14mm were further assayed for MIC and MBC  MIC (μg/mL) and MBC (μg/mL) by 2-fold dilution:  Compound 3:  17 ± 0.2mm-18 ± 0.6mm;  Compound 5:  14 ± 1.2mm -18 ± 0.5mm;  MIC and MBC for 3 and 5: 7.81μg/mL | (Fu et al., 2013) |
|  |  | Watasemycin A (3) | Thiazostatins |  |  |
|  |  | Pulicatin G (4) and  aerugine (5) | Benzyl thiazole and thiazoline |  |  |
| *Streptomyces* sp. C34 | Terrestrial (Chile): Desert Soil | Chaxamycins A-D (1-4) | Ansamycin | MIC (μg/mL):  0.13μg/mL – 0.25μg/mL;  One strain MIC:  > 32μg/mL | (Rateb et al., 2011) |
| *Streptomyces* sp. MJ635-86F5 | Terrestrial (Japan): Soil | Cremimycin | Macrolactam | MIC using serial agar dilution:  0.39μg/mL - 0.78μg/mL | (Igarashi et al., 1998) |
| *Streptomyces caelestis* | Marine (Saudi Arabia): Coastal water of red Sea | Polycyclic compounds: Compound 1 (A): Citreamicin A;  Compound 2 (B): Citreamicin B; Compound 3 (C):  Citreaglycon A (3);  Compound 4 (D): Dehydrocotreaglycon A (4) | Polycyclic xanthone | MIC (μg/mL):  Citreamicin A and citreamicin B: 0.25μg/mL;  Citreaglycon: 8μg/mL;  Dehydrocotreaglycon A:  > 50μg/mL | (Liu et al., 2012) |
| *Streptomyces* sp. A42983 | Marine (NA): NA | Gargantulide A | Macrolactone | MIC (μg/mL): 2μg/mL  Presence of serum 40%: 1μg/mL  Mice: Precluded from further study due to high toxicity in mice | (Rho et al., 2015) |
| *Streptomyces* sp. MJ929 | Terrestrial (Japan): Soil | Heliquinomycin | Benzoquinone | MIC (μg/mL) by serial agar dilution:  < 0.05μg/mL - 0.1μg/mL;  Mice: No toxicity,  LD_50_ at 100mg/kg i.p admin | (Chino et al., 1996) |
| *Streptomyces rishiriensis* MJ773-88K5 | Terrestrial (Japan): Soil | Lactonamycin (1) | Hexacyclic lactonamycinone | MIC (μg/mL) by serial agar dilution:  0.39μg/mL - 0.78μg/mL;  1 strain had MIC: 1.56μg/mL | (Matsumoto et al., 1999 |
| *Streptomyces* sp. RJA 2961 | Marine (Canada): Sediment | Ethyl acetate extract | NA | Disc diffusion: NA | (Dalisay et al., 2013) |
|  |  | Compound 1: Novobiocin 1; Compound 2: Desmethyldescarbamoylnovobiocin  Compound 3: Desmethylnovobiocin;  Compound 4:  5-Hydroxynovobiocin;  Compound 5: Desmethyldescarbamoyl-5-hydroxynovobiocin;  Compound 6:  Desmethyl-5-hydroxynovobiocin | Novobiocin | MIC (μg/mL) by microbroth dilution:  Compound 1: 0.25μg/mL;  Compound 2: > 64μg/mL;  Compound 3: 16μg/mL;  Compound 4: 8μg mL;  Compounds 5 and 6: > 64μg/mL  Known mechanism of action:  Target DNA gyrase by binding to β-subunit |  |
| *Streptomyces* sp. N1-78-1 | Marine  (Puerto Rico): Cyanobacteria | Bisanthraquinone BE-43472B (1),  BE-43472A (2),  BE-43472D (3), Bisanthraquinone: BE-43472C (4) | Bisanthraquinone | MIC and MBC (μM):  Compound 1:  0.11 μM - 0.45 μM;  Compound 2: 0.44 μM - 0.88 μM;  Compound 3: 7.1 μM - 14 μM;  Compound 4: 0.86 μM - > 55 μM;  Compound 5: Is less active than parent compound 1;  Compound 6: Not active;  Compounds 1 and 8:  Potent MBC at 0.91-3.6μM and 0.91μM respectively  MIC (IC_50_):  Compounds 1 and 6 (sufficient yield to run test) were selected Compound 1:  0.23 μM (0.11-0.90);  Compound 6:  0.45 μM (0.23-0.91) | (Socha et al., 2006b) |
|  |  | Semi-synthetic derivative compounds:  Compound 5:  Dehydrated product of 1;  Compound 6:  Dehydrated product of 2;  Compound 7:  C-12 oxidation product of 5; Compound 8:  Primary alcohol derivative of compound 1 |  |  |  |
|  |  |  |  | Time-Kill assay:  Compounds 1 and 6: Significant bactericidal activity at 4X MIC;  log_10_ CFU/mL after 24 hours antibiotic exposure:  Compound 1:  -3.17 ± 0.424;  Compound 6:  -2.98 ± 0.104  No sign of drug resistance developing. Observed as no change in MIC over 24 hours |  |
| *Streptomyces* sp. N1-78-1 | Marine  (Puerto Rico): Cyanobacteria | Bisanthraquinones 1 and 2;  Compound 3:  Dehyration product of compound 1 | Bisanthraquinone | IC_50_ (μM) by microbroth dilution:  Compound 1: 0.15μM  Compound 2: 0.36μM  Compound 3: 31μM | (Socha et al., 2006a) |
| *Streptomyces zhaozhouensis*  CA-185989 | Marine (Guinea): Sediment | New Ikarugamycins:  Compound 1: Isoikarugamycin;  Compound 2:  28-N-methylikarugamycin;  Compound 3:  30-oxo-28-N-methyl-ikarugamycin;  Compound 4: Ikarugamycin;  Compound 5: MKN-003B;  Compound 6:  1 H-indole-3-carboxaldehyde;  Compound 7: Phenylethanoic acid | Compounds 1- 4: Pentacyclic tetramic acid macrolactams; Compound 5: Butenolide; Compound 6: Indole;  Compound 7:  Acetic acid | MIC (μg/mL):  Compound 1:2- 4μg/mL Compound 2: 1- 2μg/mL Compound 3:  32- 64μg/mL;  Compound 4:  2- 4μg/mL;  Compounds 5-7:  > 64μg/mL | (Lacret et al., 2015) |
| *Streptomyce*s sp. MK277-AF1 | Terrestrial (Japan): Soil | Polyketomycin | Tetracyclic quinone glycoside | MIC (μg/mL):  0.025μg/mL - 0.2μg/mL;  Mice (i.p, mg/kg):  Acute toxicity (LD_50_):  6.25 -12.5mg/kg | (Momose et al., 1998) |
| *Streptomyces* sp. AT37 | Terrestrial (Algeria): Desert soil | Isolates | NA | Cross-streak:  26 ± 1.15 mm – 30 ± 1.00mm | (Driche et al., 2017) |
|  |  | Partially pure TLC fraction | NA | Method NA: 30mm |  |
|  |  | AT37-1 compound which is 5-[(5E,7E,11E)-2-hydroxy-9,11-dimethyl-5,7,11-tridecatrien-1-yl]-4-methyl-3(2H)-furanone | Furanone | MIC (μg/mL) by agar dilution method:  20μg/mL – 30μg/mL |  |
| *Streptomyces nashvillensis* MJ885-Mf8 | Terrestrial (Japan): Soil | Tetrodecamycin (1) and dihydrotetrodecamycin (2) | Tetronate | MIC (μg/mL) by serial agar dilution:  Compound 1: 12.5μg/mL; Compound 2: >100 μg/mL | (Tsuchida et al., 1995) |
| *Streptomyces platensis*  TP-A0598 | Marine (Japan): Sea water | TPU-A:  30-demethyllydicamycin;  TPU-B:  14,15-dehydro-8-eoxylydicamycin;  TPU-C:  30-demethyl-8-deoxylydicamycin;  TPU-D:  8-deoxylydicamycin | Lydicamycin | MIC (μg/mL) using 2-fold dilution:  TPU-A: 12.5μg/ml;  TPU-B: 12.5μg/ml;  TPU-C: 3.13;  TPU-D: 3.13μg/ml, 12.5μg/ml;  Lydicamycin 6.5μg/mL | (Furumai et al., 2002) |
| *Streptomyces griseus* M-33-5 | Terrestrial (Turkey): Soil | NA | NA | Agar plaque technique:  M-33- 5 is the most active | (Urgen et al., 2010) |
|  |  | 4’-deacetyl griseusin A (1) and griseusin A (2) | Pyranonapthoquinone | MIC (μg/mL) by microbroth dilution:  Ethyl acetate extract: 0.75μg/mL ;  Compound 1: 0.5μg/mL;  Compound 2: 1.0μg/mL |  |
| *Streptomyces* sp. CAH29 | Terrestrial (Turkey): Rhizosphere soil of *Achillea ketenoglui* | Tetrangomycin | Anthraquinone | Disk diffusion:  12mm tetrangomycin/  22mm Ethyl acetate extract  Docking study of tetrangomycin:  Inhibitor 830  Van der Waals’:  - 30.93: - 46.96  Electrostatic: -2.78: - 2.59  Total: -33.71: - 49.55 | (Özakin et al., 2016) |
| *Streptomyces hygroscopicus* var. *azalomyceticus* | NA | Azalomycin F5a (1) and its 4 derivative compounds: NA | Polyhydroxy macrolide | Agar dilution method:  Compound 2: 23.1mm;  Compound 3: 22.8mm;  Compound 4: 23.3mm;  Compound 5: 23.5mm  MIC (μg/mL):  Compound 1: 3- 4μg/mL; Compound 2: 0.5- 1μg/mL; Compound 3: 0.67- 1μg/mL; Compound 4:  0.67- 0.83μg/mL;  Compound 5: 0.5- 0.83μg/mL | (Yuan et al., 2014) |
| **NRPS** | | | | | |
| *Streptomyces* sp. HW-003 | Terrestrial (Korea): Soil | Peptide AMRSA1 with MW: 1100 containing alanine and serine | NA | Disc diffusion: Produced ZOI  MIC (μg/mL) by 2-fold serial agar dilution: 0.01μg/mL - 0.1μg/mL | (Lee et al., 1997) |
| *Streptomyces capillispiralis* MTCC10471 | Marine (India): Sediment | Actinomycin D | Chromopeptide | Agar overlay technique:  Produced ZOI  MIC (μg/mL): 1μg/mL | (Srinu eFSUt al., 2013) |
| *Streptomyces* sp. CNT-373 | Marine (Fiji): Sediment | Nosiheptide | Thiopeptide | MIC (μg/mL) by agar dilution: 0.03 - 0.125mg/L  Time-kill kinetics:  Rapid bactericidal in a concentration and time-dependent manner;  At 10X and 20X MIC showed nearly 2-log kill in 6 hours  PAE:  Prolonged PAE exceeding 9 hours  Presence of 20% serum: MIC unaffected when tested against USA300 MRSA;  *in vivo* mice (20mg/kg, i.p): Compound provided significant protection against mortality | (Haste et al., 2012) |
| *Streptomyces fulvissimus* MTCC 7336 | Freshwater (India): Soil | A high molecular weight peptide of 63kDa | Glycopeptide | Disc diffusion:  19.00 ± 1.0mm | (Malik et al., 2008) |
| *Streptomyces zagrosensis* UTMS 1154 | Terrestrial (Iran): Soil | Persipeptides A and B | *N*-methylated cyclic persipeptide | Disc diffusion: 15mm | (Mohammadipanah et al., 2016) |
| *Streptomyces* sp. CNS-575 | Marine (Fiji): Sediment | Fijimycins A-C (1-3) | Cyclic depsipeptides | MIC_100_ (μg/mL):  Fijimycin A: 4μg/mL, 16μg/mL, 3 μg/mL;  Fijimycin B: > 32μg/mL;  Fijimycin C: 8μg/mL, > 32μg/mL | (Sun et al., 2011) |
| *Streptomyces* sp. SUK 25 | Terrestrial (Malaysia): Endophyte | Cyclo-(tryptophanyl-prolyl) and chloramphenicol | Cyclic diketopiperazine dipeptides and amphenicol | Cross streak: NA  Disk diffusion, MIC (μg/mL) and MBC (μg/mL):  FI: 14 mm; MIC: 16μg/mL; MBC: 128μg/mL;  FII: 15 mm; MIC: 8μg/mL MBC: 32μg/mL;  FIII-VI, FVIII: NA;  FXI-FXIII: NA;  FVII: 22mm; MIC: 8μg/mL; MBC: 64μg/mL;  FVII-F2: 15mm; MIC: 16μg/mL; MBC: 32μg/mL;  FVII-F3: 16mm; MIC: 8μg/mL; MBC: 64μg/mL;  FX: 14mm; MIC: 16μg/mL; MBC: 128μg/mL;  FIX: 13mm; MIC:16μg/mL; MBC: 128μg/mL | (Alshaibani et al., 2016) |
| *Streptomyces alboflavus* 313 | Terrestrial (China): Mountain Soil | NW-G08 and  NW-G09 | Cyclic hexapeptide | MIC (μg/mL) by microbroth dilution:  NW-G08: 1.56μg/mL; NW-G09: 12.5 μg/mL | (Ji et al., 2012) |
| *Streptomyces alboflavus* 313 | Terrestrial (China): Mountain Soil | NW-G10 (1) and  NW-G11 (2) | Piperazic acid-containing cyclic hexapeptides | MIC (μg/mL) by microbroth dilution:  Compound 1: 6.25μg/mL;  Compound 2: 25.0μg/mL | (Wei et al., 2012) |
| *Streptomyces* sp. HIL Y-9420704 | Terrestrial (Greece): Soil | Methylsulfomycin I | Cyclic Thiopeptide | MIC (μg/mL): 0.06μg/mL - 0.125μg/mL against several *Staphylococcus* and *Enterococcus* species  *in vivo* mice:  At 25mg/kg in mice were not conclusive due to precipitation | (Vijaya Kumar et al., 1999) |
| *Streptomyces* sp. IMB 094 | Marine (China): Sediment | Neo-actinomycin A and B, actinomycin D and actinomycin X2 | Chromopeptide lactone | MIC (μg/mL) by agar dilution:  Neo-actinomycin A: 16μg/mL, 64μg/mL;  Neo-actinomycin B: > 128μg/mL;  Actinomycin D and Actinomycin X2: 0.25μg/mL | (Wang et al., 2017) |
| *Streptomy*ces sp. K04-0144 | Terrestrial (Japan): Soil | Nosokomycin A (1) -D (4) | Moenomycin | Disk diffusion:  Nosokomycin A: 27mm; Nosokomycin B: 25mm; Nosokomycin C: 23mm;  Nosokomycin D: 22mm;  MIC (μg/mL) by  microbroth dilution:  Compounds 1-4: 0.25μg/mL  Silk worm *in vivo* mimic Silkworm assay:  Silkworms survived > 3 days when nosokomycin A or B was administered at a dose of 50µg/larva | (Uchida et al., 2010) |
| *Streptomyces amritsarensis* sp. nov. | Terrestrial (NA): Soil | Amino acid sequence as Ala-Thr-Gly-Ser-His-Gln and a long chain fatty acid tail with six times repeated the molecular mass of 161 Da which is corresponding to -C12H19.  Molecular mass (878.5 Da) | Lipopeptide | Disk diffusion: 13mm;  MIC (μg/mL) by microbroth dilution: 45μg/mL | (Sharma et al., 2014) |
| *Streptomyces* sp. MI982-63F1 | Terrestrial (Japan): Soil | Vinylamycin | Depsipeptide | MIC (μg/mL) by serial agar dilution:  3.13 μg/mL and 6.25μg/mL  *in vivo* mice: No acute toxicity at 100mg/kg, i.p | (Igarashi et al., 1999) |
| **Others** | | | | | |
| *Streptomyces* sp. MC11024 | Terrestrial (Japan): Soil | Streptorubin B | Prodiginine | MIC (μg/mL) by 2-fold dilution:  ≥ 32μg/mL;  Anti-biofilm (μg/mL):  1μg/mL:  Reduced biofilm to 27%, however cell growth not affected;  IC_50_:  0.22μg/mL (0.56μM) | (Suzuki et al., 2015) |
| *Streptomyces* sp. VITBRK1 | Marine (Bay of Bengal): Sediment | Supernatant | Indole | Disk diffusion: 13mm, 21mm, 21mm, 24mm | (Rajan and Kannabiran, 2013a) |
|  |  | Indolo compound from the ethyl acetate extract |  | NA: NA |  |
| *Streptomyces* sp. VITBRK3 | Marine (India): Sediment | 2,4-dichloro-5-sulfamoyl  benzoic acid | Benzoic acid | MIC_50_ (μg/mL):  4.0 ± 0.5μg/mL and  1.8 ± 0.14μg/mL | (Rajan and Kannabiran, 2015) |
| *Streptomyces parvulus* C1.192 | Terrestrial (Brazil): rhizosphere of the plant *Caesalpinia pyramidalis* Tul. | Isolates | NA | Agar blocks:  C1.192 most active | (Silva-Lacerda et al., 2016) |
|  |  | Supernatant | NA | Disc diffusion:  28+ 0.81mm in media MPE at 72 hours;  28+ 0.81mm in ISP- 4 media at 48 hours;  28mm in ISP- 4 at 72 hours |  |
|  |  | Ethanol extract of biomass |  | MIC (μg/mL):  0.97μg/ mL)  MBC (μg/mL):  1.95μg/mL |  |
|  |  | Ethyl acetate extract of liquid metabolite |  | MIC (μg/mL): 3.9μg/mL;  MBC (μg/mL): 31.25μg/mL |  |
| *Streptomyces* sp. KBI TISTR 2304 | Air sample (Thailand) | 2-4-di-tert-butylphenol | Butylphenol | MIC (μg/mL): 31.25μg/mL (15.63 μg/mL- 62.50μg/mL);  MBC (μg/mL): 31.25μg/mL (15.63μg/mL - 62.50μg/mL) | (Chawawisit et al., 2015) |
| *Streptomyces*  sp. KB1 TISTR 2304 | Air Sample (Thailand) | 2,4-Di-tert-butylphenol in presence of vancomycin | Butylphenol | MIC using 2-fold microbroth serial dilution:  2,4- Di-tert-butylphenol:  15.63μg/mL – 62.50μg/mL;  Vancomycin:  1.56 μg/mL- 3.12μg/mL; | (Chawawisit et al., 2016) |
|  |  |  |  | FIC value using 2-fold serial dilution:  2,4-Di-tert-butylphenol 0.56 - 1.5  Vancomycin: 0.06-1.0  FIC Index: 0.56- 1.5  Activity: Partial synergism (0.5 ≤ FIC index < 0.76) - Indifferent activity (1.0 ≤ FIC index < 4.0) |  |
| *Streptomyces* sp. CNQ-525 | Marine (USA): Sediment | Napyradiomycin A80915A and A80915B | Meroterpenoid dihydroquinone | MIC (μg/mL) by microbroth dilution:  Napyradiomycin A80915A:  1μg/mL, 1- 2μg/mL, 2μg/mL, 1.5-3 μg/mL, 3μg/mL;  Napyradiomycin A80915B:  1μg/mL, 1.5 μg/mL, 2μg/mL, 1-3 μg/mL | (Haste et al., 2011a) |
|  |  |  |  | Bactericidal for compound 1 and 2 at 4 hours compared;  Complete killing at 2 hours for 10X Compound 2 demonstrated more potent killing with 4-log decrease in bacterial counts at 2 hours in 1X MIC |  |
|  |  |  |  | 20% normal human serum: Diminished anti-MRSA activity |  |
| *Streptomyces* sp. BCNU 1001 | Terrestrial (Korea): Forest soil | Isolates | NA | Cross streak and agar overlay:  No MRSA strain tested at this stage | (Choi et al., 2012) |
|  |  | Ethyl acetate extract |  | MIC (mg/disc): 0.5mg/disc - 1mg/disc |  |
|  |  | 2-hydroxybenzyl alcohol as the most abundant compound isolated | Benzyl alcohol | MIC (μg/mL):  62.5μg/mL, 125μg/mL |  |
| *Streptomyces* sp. K04-0144 | Terrestrial (Japan): Soil | Cyslabdan | Labdane-type diterpene | Disc diffusion of cyslabdan: No activity | (Fukumoto et al., 2008) |
|  |  |  |  | MIC (10μg/disk) of cyslabdan: Weak activity at 64μg/mL |  |
|  |  |  |  | MIC of imipenem in presence of cyslabdan (10μg/mL):  Potentiated imipenem with reduction from 16μg/mL to 0.015μg/mL (1070 - fold potentiation) against K24 MRSA (1 strain); |  |
|  |  |  |  | MIC of imipenem in presence of cyslabdan (20μg/mL): MIC: Potentiated imipenem with reduction from 16μg/mL - 64μg/mL to 0.125μg/mL - 64μg/mL (22 MRSA strains);  MIC_50_: Potentiated imipenem with reduction from 32μg/mL - 0.25μg/mL (22 MRSA strains);  MIC_90_: Potentiated imipenem with reduction from 64μg/mL - 32 μg/mL (22 MRSA strains);  MIC of β-lactams in presence of cyslabdan:  Cyslabdan potentiated the anti-MRSA activity of all β-lactams tested. In particular reduced the activity of carbapenems (128-1070 fold) |  |
| *Streptomyces* sp. K04-0144 | Terrestrial (Japan): Soil | Cyslabdan | Labdane-type diterpene | MIC (μg/mL):  Imipenem and cyslabdan led to reduced MIC of imipenem: 0.015 μg/mL;  Mechanism of action:  Cyslabdan targets the Fem A enzyme responsible for synthesis of peptidoglycan cell wall | (Koyama et al., 2012) |
| *Streptomyces* sp. CNQ-329, *Streptomyces* sp. CNH-070, | Marine (USA): Sediment | Napyradiomycins 1- 6 are new derivatives;  Napyradiomycins 7- 9 known compounds Napyyradiomycin B2 (7)-B4 (9) | Meroterpenoid dihydroquinone | MIC (μg/mL):  Napyradiomycin 1:  16μg/mL;  Napyradiomycin 2:  64μg/mL;  Napyradiomycin 3- 6:  > 64μg/mL;  Napyradiomycin B2:  32- 64μg/mL;  Napyradiomycin B3:  2 μg/mL;  Napyradiomycin B4:  32μg/mL | (Cheng et al., 2013b) |
| *Streptomyces* sp. CNQ-418 | Marine (USA): Sediment | Marinopyrrole A;  16 other derivatives of 1,3 -bipyrrole | 1,3 -bipyrrole | MIC (μg/mL) by macrobroth dilution:  0.188- 0.375μg/ mL, 0.375μg/mL | (Haste et al., 2011b) |
|  |  |  |  | Time-kill assay using rapid and concentration-dependent bactericidal killing;  Within 9 hours at 10X MIC showed 2- log kill, at 20X MIC reduced initial inoculum by early 6-log. ie marinopyrrole killed faster at 9 hours than vancomycin and linezolid tested at same concentration and time |  |
|  |  |  |  | PAE (μg/mL):  Favourable pharmacological profile showing prolonged PAE at both 10X MIC and 20X MIC |  |
|  |  |  |  | Resistance studied via serial-passage mutagenesis:  Exhibited sustained potent inhibitory activity despite repeated bacterial passage in sub-MIC doses of compound |  |
|  |  |  |  | Presence 20% of human serum:  ≥ 96 μg/mL (256-fold higher) and may be due to high protein binding |  |
|  |  |  |  | Adsorption to plastic:  Amount that adsorbed to plastic increased in linear fashion with increase in MIC from 1X, 2X, 5X and 10X MIC. Glass tube did not retain marinopyrrole at any of the tested concentrations. |  |
|  |  |  |  | Derivatives in the presence of 20% human serum:  Increased MIC in presence of 20% human serum |  |
| *Streptomyces.* sp. CNQ-418 | Marine (USA): sediment | Marinopyrrole A, marinopyrrole B, marinopyrrole C, marinopyrrole D, marinopyrrole E, marinopyrrole F | 1,3'- Bipyrrole | MIC_90_ (μg/mL):  Marinopyrrole A: 0.31μg/mL Marinopyrrole B: 0.63μg/mL Marinopyrrole C: 0.16μg/mL Marinopyrrole F: 3.1μg/mL;  Compound 11: 1.6μg/mL;  Compounds 12- 13: NSA (No significant activity; > 8μg/mL;  Compound 14: 0.78μg/mL;  Compound 15: 6.3μg/mL;  Compound 16: 1.6μg/mL | (Hughes et al., 2010) |
|  |  | Synthetic derivatives  Compound 11:  4,4’,5,5’-Tetrachloro-1’-H-  1,3’-bipyrrole-2,2’-diyl) bis((2-acetoxyphenyl)methanone);  Compound 12:  (4,4’,5,5’-Tetrachloro-1’-methyl-1’-H-1,3’-bipyrrole-2,2’-diyl)-  bis ((2-methoxyphenyl)methanone);  Compound 13:  (4,4’,5,5’-Tetrachloro-1’-methyl-1’H-1,3’-bipyrrole-2,2’-diyl)-bis((2-hydroxyphenyl)methanone);  Compound 14:  (4,4’,5’-Trichloro-5-methoxy-1’H-1,3’-bipyrrole-2,2’-diyl)bis-((2-hydroxyphenyl methanone);  Compound 15:  N-(2-(3,4’,5’-Trichloro-2’,5-bis(2-hydroxybenzoyl)-1’H-1,3’-bipyrrol-  2-ylthio)ethyl)acetamide;  Compound 16:  (4,4’,5’-Trichloro-5-(dimethylamino)-1’H-1,3’-bipyrrole-2,2’-diyl)bis(2-hydroxyphenyl)methanone |  |  |  |
| *Streptomyces.* sp. CS684 | Terrestrial (Korea): Soil | Laidlomycin | Polyether ionophores | MIC (μg/mL) by agar dilution: 1μg/mL | (Jin et al., 2007) |
| *Streptomyces* sp. 04DHS2 | Marine (Korea):  Sediment | 1-actetyl-ß-Carboline | β-Carboline | MIC (μg/mL) by 2-fold serial dilution:  64μg/mL | (Shin et al., 2010) |
|  |  |  |  | MIC (μg/mL) of active compound in combination with ampicillin:  At 32 μg/mL: 8μg/mL, 16μg/mL  FIC index:  0.156, 0.250, 0.281, 0.188;  At 64 μg/mL: 4μg/mL, 8μg/mL;  FIC index: 0.266, 0.313, 0.516, 0.281 |  |
|  |  |  |  | MIC (μg/mL) of active compound in combination with penicillin:  At 32μg/mL: 16μg/mL, 32μg/mL  FIC index: 0.188, 0.250, 0.375, 0.313;  At 64 μg/mL: 8μg/mL, 16μg/mL  FIC index: 0.281, 0.313, 0.375;  MIC (μg/mL) of active compound in oxacillin  At 32μg/mL: 64μg/mL, 128 μg/mL, 256μg/mL, 512 μg/mL;  FIC index: 1.125, 0.625, 1.250, 0.375  At 64μg/mL: 64μg/mL, 128μg/mL, 256μg/mL  FIC index: 0.750, 1.250, 1.500, 0.500 |  |
| *Streptomyces albospinus*  15-4-2 | Terrestrial (China): Soil | Compound 1:  4α,10β-dimethyl-decahydronaphthalene-1β,3β,5α,8α-tetraol;  Compound 2:  5β H-eudesmane-1β,6α,11-triol | Sesquiterpene | Disc diffusion:  Compound 1: 10.0 mm  Compound 2: NA | (Yu et al., 2013) |
| *Streptomyces* sp. CS392 | Terrestrial (Korea): Soil | Supernatant culture broth | NA | Disc diffusion: Media optimization:  Carbon sources: Glucose and mannitol, 24 hours, ca. 17mm  Nitrogen sources: Peptone and beef, 24 hours, ca.16mm  Minerals: KCl, MgCl_2_: 12 hours, 18mm | (Cho et al., 2012) |
|  |  | C1 (2.4mg), Rf : 0.4;  C2 (6.3mg) Rf : 0.5;  C3 (12.3mg) Rf : 0.6 | NA | MIC (μg/mL) by agar dilution:  C1 and C2: 4.06μg/mL;  C3: 2.03 μg/mL |  |
| *Streptomyces* sp. SJY056 | Freshwater (China): Soil | Isolates | NA | Confrontation plate assay: Selected based on ZOI formed | (Zhu et al., 2013) |
|  |  | Organic phase of ethyl acetate extract |  | Disc diffusion: 21mm |  |
|  |  | Inorganic phase |  | No activity |  |
| *Streptomyces* sp. KEH23 | Terrestrial (Cyprus): Soil | Isolates | NA | Cross streak: Selected based on ZOI | (Oskay, 2009) |
|  |  | Supernatant of culture broth |  | Agar well diffusion: 22 mm |  |
| *Streptomyces* sp. SUK 25 | Terrestrial (Malaysia): Endophyte from *Zingiber spectabile* | Isolates | NA | Agar blocks: 20mm, 21mm and 30mm | (Junaidah et al., 2015) |
|  |  | Methanolic extract |  | Disk diffusion (2mg/mL): Pour plate method:19 ± 0.26mm;  Spread plate method:  15 ± 0.38mm |  |
|  |  | Ethyl acetate extract |  | MIC (μg/mL)  2.44 ± .01μg/mL in Thronton’s media, pH 7, 7 days fermentation, 400mL  0.975 ± 0.001μg/mL in Thronton’s media, pH 7, 7 days fermentation, 400mL with aeration rate of 140rpm |  |
| *Streptomyces* sp. SUK 25 | Terrestrial (Malaysia): Endophyte from *Zingiber spectabile* | Ethyl acetate extract | NA | MIC (μg/mL): 1.95μg/mL | (Ahmad et al., 2015) |
|  |  |  |  | Time-kill assay:  Showed concentration dependent bacteriostatic action.  1X MIC 1.95μg/mL,  2X MIC; 9μg mL,  4X MIC 7.8μg/mL  8X MIC 15.6μg/mL |  |
|  |  |  |  | Cell lysis assay:  Cell lysis dropped to 50% at 127.5 min compared to vancomycin at 105min, untreated cells at 172.5 min  Total cell lysis at 7.8 μg/mL compared to vancomycin 6.25 μg/mL |  |
|  |  |  |  | Crystal violet uptake assay:  Extract caused irregular shape of cells which affected uptake of crystal violet  Uptake reduced to 47.62% compared to untreated cells (58.73%) |  |
|  |  |  |  | Release of UV materials:  Release of UV absorbing materials at 260nm and 280nm was due to cell lysis  Release at 260nm: 0.073 ± 0.02;  Release at 280nm: 0.074 ± 0.080 |  |
| *Streptomyces* sp. R3YS, *Streptomyces* sp. 2A, *Streptomyces* sp. N23, *Streptomyces* sp. A26, *Streptomyces* sp. A27, *Streptomyces* sp. A13 | Terrestrial (India): Soil | Isolates | NA | Agar blocks:  R3YS 21 ± 0.5 mm - 24 ±1.0mm | (Sharma et al., 2011) |
|  |  | Supernatant |  | Agar well diffusion: NA |  |
|  |  | n-butanol extract |  | Diffusion:  R3YS: 24 ± 1.0mm, 21 ± 0.5mm, 24 ± 0.0mm;  2A: 21.6 ± 0.5mm, 19.3 ± 0.5mm, 19.3 ± 0.5mm  N23: 17±1.0 mm, 14.6 ± 0.5mm, 15 ± 0.5mm;  A27: 17 ± 0.0, 16 ± 1.0mm, 14.6 ± 0.5mm  A13: 17 ± 0.0mm, 16 ±1.0mm, 14.6 ± 0.5mm |  |
|  |  | Diethyl ether extract |  | Diffusion:  A26: 18.3 ± 0.5, 15.6 ± 0.5mm, 15.6 ± 0.5mm, 15.6 ± 0.5mm |  |
| *Streptomyce*s sp. N II 1054, *Streptomyces* sp. NII 716, *Streptomyces* sp. NII 714 | Terrestrial (India): Soil | Methanolic extract | β-lactamase inhibitor of *B. cereus* | Agar well diffusion:  N II 1054,  NII 716 and  NII 714 showed ZOI | (Mohandas et al., 2012) |
|  |  |  |  | Anti-MRSA by agar well diffusion:  N II 1054:  23 ± 0.5mm;  NII 716: NA;  NII 714:  10 ± 1mm |  |
| *Streptomyces albus* ICN33 | Marine (India): Sponge | Extracts: Hexane: chloroform, chloroform: ethyl acetate, ethyl acetate: methanol, methanol | NA | Disc Diffusion:  10- 20mm | (Iniyan et al., 2016) |
|  |  | Compound PVI331: 506.2636 Da,  Rf: 0.45,  Retention time of 29.210 and 29.339 at 246.5nm UV absorbance |  | MIC (μg/mL) by microbroth dilution: 1μg/mL  Time Kill assay: At 4X MIC cell counts decreased at 2 hours and 4 hours  Anti-biofilm:  Carried out on *S. aureus* strain, not MRSA strain |  |
| *Streptomyces* sp. ICN 12, *Streptomyces* sp. ICN14, *Streptomyces* sp. ICN 15, *Streptomyces* sp*.* ICN18, *Streptomyces* sp. ICN20, *Streptomyces* sp. ICN23, *Streptomyces* sp. ICN26, *Streptomyces* sp. ICN 31, *Streptomyces* sp. ICN34 , *Streptomyces* sp. ICN 5 | Marine (India): Mangrove rhizosphere soil | Isolates | NA | Double layer agar assay:  14 active isolates including 10 *Streptomyces* isolates | (Iniyan et al., 2017) |
|  |  | Ethyl acetate extract |  | Disc diffusion:  ICN5, ICN18, ICN23, ICN31, ICN 34: > 20mm ICN12, ICN 14, ICN15, ICN20, ICN 26: 11- 20mm |  |
|  |  | ICN 5: Rf 0.81, Retention time: 0.712, 0.935, 1.273, 1.662, 2.315 (UV 215nm) | Phytochemical: Alkaloids, flavonoids, steroids, terpenoids | MIC (μg/mL):  ICN5: 8μg/mL;  ICN12: 32μg/mL  ICN14: 16μg/mL;  ICN15: 32μg/mL  ICN18: 12μg/mL;  ICN 20: 16μg/mL  ICN23: 4 μg/mL;  ICN 26: 32 μg/mL  ICN 31: 4 μg/mL;  ICN 34: 16 μg/mL  Time-Kill assay:  Not a single isolate was bactericidal at 2 hours;  ICN 23 and ICN31 were found to have bactericidal action at 8 hours  3 log reduction of visibility of tested 5 fractions were observed after 24 hours of exposure |  |
|  |  | ICN 18: Rf 0.69, Retention time: 2.160, 3.812, 6.073, 6.862 (UV 240, 250, 273 nm) | Phytochemical: Alkaloids, flavonoids, saponins, steroids, phenolics, terpenoids |  |  |
|  |  | ICN 23: Rf 0.86, Retention time: 3.233, 3.375 (UV 210nm) | Phytochemical: Alkaloids, flavonoids, phenolics, steroids, tannins, terpenoids |  |  |
|  |  | ICN 31: 0.65 Rf; Retention time: 2.002,5.321, 6.584, 7.760, 10.767, 12.264, 13.310, 15.355 (UV 240 250, 256nm) | Phytochemical: Alkaloids, flavonoids, glycosides, phenolics, steroids, tannins, terpenoids |  |  |
| *Streptomyces* sp. SLO-105 | Freshwater (Algeria): Lake sediment | Isolate | NA | Double layer agar assay: 60mm | (Morakchi et al., 2009) |
| *Streptomyces* sp. VITBRK 2 | Marine (India): Sediment | Supernatant | NA | Agar plate diffusion: 12mm - 21mm | (Rajan and Kannabiran, 2014) |
| *Streptomyces* sp. MUSC 135^T^ | Marine (Malaysia): Intertidal soil | Bacteriocin | NA | NA:NA | (Ser et al., 2015d) |
| *Streptomyces* sp. IMD 2703 | Terrestrial (Cambodia): Soil | Partially purified compound from combination of charcoal, ion-exchange and dialysis | NA | Agar diffusion: 12mm- 22mm | (Higginbotham and Murphy, 2010) |
| *Streptomyces* sp. SUK 06 | Endophyte of *Thottea grandiflora* (Malaysia) | Ethyl acetate extract | NA | Agar plug: 37mm | (Ghadin et al., 2008) |
| *Streptomyces* sp. PVRK-1 | Marine (India): Mangrove | Isolates | NA | Double layer assay:  Optimization: Tryptone 50 as N source: 25mm  pH 7: 25mm  37ºC: ca. 26mm  1% NaCl: ca. 23 mm | (Kannan et al., 2011) |
|  |  | A2 molecule with Rf: 0.37, Retention time: 1.389 (UV 270nm) |  | MIC (μg/mL) by microbroth dilution: 30μg/mL  MIC of infected Zebrafish embryos: 32-34μg/mL |  |
| *Streptomyces* sp. CNQ-509 | Marine (USA): Sediment | Nitropyrrolin A-E (1-5);  3-farnesylpyrrole;  γ-farnesyl-R-nitropyrrole;  2-farnesylpyrrole | Sesquiterpene and synthetic pyrrole | MIC (μg/mL):  No significant activity (≥ 20μg/mL) observed for all compounds except one of the synthetic derivative (3-farnesylpyrrole: 2.8μg/mL) | (Kwon et al., 2010) |
| *Streptomyces rubrolavendulae* ICN3 | Marine (India): Rhizosphere soil region of the mangrove *Avicennia officinalis* | Isolates | NA | Double layer agar assay:  21 mm but in the nutrient optimized culture conditions ZOI:  43 ± 1.53 mm | (Kannan et al., 2014) |
|  |  | C23 | Retention time is 2.062 min | MIC (μg/ mL) by microbroth dilution: 2.5μg/mL |  |
|  |  |  |  | *in vivo* Zebrafish embryos:  Infected embryos survived in presence of 5μg/mL of C23 |  |
| *Streptomyces* sp. 1492 | Terrestrial (Turkey): Karstic cave Soil | Isolates | NA | Agar piece method:  90 actinomycetes, 180 isolates were active against all 10 strains; | (Yücel and Yamaç, 2010) |
|  |  |  |  | From 180, 27 %: Gram-negative only; 33%: Gram-positive only |  |
|  |  | Compound with Rf: 0.72 | NA | Disc diffusion  Strain 105: 10mm Strain 320, 404 and 1613: - Strain 1421: 8mm Strain 1492: 15mm Strain 1910: 8.6 mm  MIC (μg/mL)  Without heat: MIC: 125 μg/mL  MBC: 1000μg/mL |  |
|  |  |  |  | With heat, 60°C, 30mins: MIC: 500  MBC: 1000μg/mL  With heat, 100°C, 5mins: MIC: 500μg/mL  MBC: 1000μg/mL |  |
| *Streptomyces pluripotens* sp. nov., | Marine (Malaysia):  Mangrove soil | Supernatant | NA | Agar well: 10.5mm | (Lee et al., 2014b) |
| *Streptomyces psammoticus* sp. BT-408 | Marine (India): Sediments | Culture filtrate | NA | Agar cup plate: 20mm | (Sujatha et al., 2005) |
|  |  | SBR-22 |  | MIC (μg/mL): 64μg/mL |  |
| *Streptomyces* sp. M10-77, *Streptomyces* sp. I-400 A, *Streptomyces* sp. B1-T61 | Marine (Peru): Sediment | Preliminary assay in spanish Language | NA | Agar overlay  M10-77: 50mm (92% inhibition) I-400A: 77mm (85.7% inhibition) B1-T61: 62m (85.2%) | (León et al., 2011) |
|  |  | Dichloromethane extract of 3 isolates | NA | Agar well:  M10-77: 40mm (87.5% inhibition) I-400A: 14mm (64.2% inhibition) B1-T61: 24mm (79.16% inhibition) |  |
|  |  | Ethyl acetate extract |  | MIC (μg/mL):  M10-77: 7.9μg/mL; I-400A: 31.7μg/mL; B1-T61: 31.7μg/mL |  |
| *Streptomyces* sp. G60 | Terrestrial (Algeria): Desert soil | Isolates | NA | Cross-streak: NA | (Driche et al., 2015) |
|  |  | di-(2ethylhexyl)phtalate | Phthalates | Disc diffusion:  26.5 ± 1.0 mm |  |
| *Streptomyces tendae* | Marine (Egypt): Soil | A β-lactamase inhibitory protein | High content in therionine (90 mole percent), arginine (75 mole percent) and alanine (70 mole percent) | Disk diffusion: 35mm of filtrate;  Chromogenic cephalosporin spot test (u):  Detection of β-lactamase activity: 180u | (Abdulkhair and Alghuthaymi, 2016) |
| *Streptomyces albofaciens* | Marine (India): Sediment | Isolates | NA | Cross streak, cylinder plates, agar plate, agar overlay, well diffusion and disc diffusion: NA | (Rajan and Kannabiran, 2013b) |
|  |  | Ethyl acetate extract |  | NA: 21 mm  MIC using double dilution and MBC (μg/mL)  MIC: 512μg/mL and MBC: 1024μg/mL |  |

NA: Data not available
